# The Redox Homeostasis of Skeletal Muscle Cells Regulates Stage Differentiation of *Toxoplasma gondii*

**DOI:** 10.3389/fcimb.2021.798549

**Published:** 2021-11-22

**Authors:** Md. Taibur Rahman, Izabela J. Swierzy, Bryan Downie, Gabriela Salinas, Martin Blume, Malcolm J. McConville, Carsten G. K. Lüder

**Affiliations:** ^1^ Institute for Medical Microbiology and Virology, University Medical Center Goettingen, Georg-August-University, Goettingen, Germany; ^2^ Department of Biochemistry and Molecular Biology, University of Dhaka, Dhaka, Bangladesh; ^3^ Transcriptome and Genome Analysis Laboratory, University Medical Center Goettingen, Georg-August-University, Goettingen, Germany; ^4^ Department of Biochemistry and Molecular Biology, Bio21 Institute of Molecular Science and Biotechnology, The University of Melbourne, Parkville, VIC, Australia; ^5^ Junior Research Group 'Metabolism of Microbial Pathogens', Robert-Koch-Institute, Berlin, Germany

**Keywords:** Toxoplasma gondii, stage conversion, skeletal muscle, redox homeostasis, host metabolism

## Abstract

*Toxoplasma gondii* is an obligatory intracellular parasite that causes persistent infections in birds and mammals including ~30% of the world’s human population. Differentiation from proliferative and metabolically active tachyzoites to largely dormant bradyzoites initiates the chronic phase of infection and occurs predominantly in brain and muscle tissues. Here we used murine skeletal muscle cells (SkMCs) to decipher host cellular factors that favor *T. gondii* bradyzoite formation in terminally differentiated and syncytial myotubes, but not in proliferating myoblast precursors. Genome-wide transcriptome analyses of *T. gondii*-infected SkMCs and non-infected controls identified ~6,500 genes which were differentially expressed (DEGs) in myotubes compared to myoblasts, largely irrespective of infection. On the other hand, genes related to central carbohydrate metabolism, to redox homeostasis, and to the Nrf2-dependent stress response pathway were enriched in both infected myoblast precursors and myotubes. Stable isotope-resolved metabolite profiling indicated increased fluxes into the oxidative branch of the pentose phosphate pathway (OxPPP) in infected myoblasts and into the TCA cycle in infected myotubes. High OxPPP activity in infected myoblasts was associated with increased NADPH/NADP^+^ ratio while myotubes exhibited higher ROS levels and lower expression of anti-oxidants and detoxification enzymes. Pharmacological reduction of ROS levels in SkMCs inhibited bradyzoite differentiation, while increased ROS induced bradyzoite formation. Thus, we identified a novel host cell-dependent mechanism that triggers stage conversion of *T. gondii* into persistent tissue cysts in its natural host cell type.

## Introduction

The intracellular parasite *Toxoplasma gondii* is widespread in birds and mammals including an estimated 30% of the world’s human population. Infections of immunocompetent hosts are mostly asymptomatic but lead to long-term parasite persistence in brain and muscle tissues. Acquired immune-suppression in chronically infected individuals or trans-placental transmission of the parasite to fetuses following primary infection can however lead to severe and life-threatening reactivated *Toxoplasma* encephalitis and congenital toxoplasmosis, respectively (Montoya and Liesenfeld, 2004). Furthermore, *Toxoplasma* infections of otherwise healthy adolescent and adults have been recognized as a significant cause of posterior uveitis (Holland, 2003). Toxoplasmosis is thus among the five most important food-borne infectious diseases in the US (Hoffmann et al., 2012) and probably also other regions in the world.

Following oral uptake of infectious sporozoites or bradyzoites and infection of the intestinal epithelium, the parasite differentiates into so-called tachyzoites which rapidly proliferate in various hematopoietic and non-hematopoietic cells and disseminate throughout the host. In immunocompetent hosts, tachyzoites induce a robust cell-mediated immune response that limits parasite growth and leads to the eradication of most parasites. However, some parasites evade killing by differentiating into slowly replicating and largely dormant bradyzoites which form long-lived tissue cysts in neurons and muscle cells. The formation of bradyzoite-containing tissue cysts is crucial for transmission of *T. gondii* to new hosts *via* ingestion of infected prey or undercooked meat. These stages are also responsible for reactivated toxoplasmosis after de-differentiation of dormant bradyzoites to lytic and tissue-damaging tachyzoites in immune-compromised patients (Montoya and Liesenfeld, 2004).

Differentiation of *T. gondii* from tachyzoites to bradyzoites is accompanied by expression and repression of multiple stage-specific proteins, metabolic reprogramming, cyst wall formation and cessation of cell cycle progression [recently reviewed in (Jeffers et al., 2018)]. Bradyzoite differentiation was shown to be regulated by a complex interplay of translational control *via* phosphorylation of the *T. gondii* initiation factor 2 subunit α (TgIF2α) (Narasimhan et al., 2008), repression and activation of gene transcription by members of the AP2 transcription factor family (Radke et al., 2013; Walker et al., 2013), cell cycle-dependent mechanisms (Radke et al., 2003), tightly regulated cAMP levels (Kirkman et al., 2001; Hartmann et al., 2013), and chromatin modifiers including TgHDAC3 and TgGCN5a (Bougdour et al., 2009; Naguleswaran et al., 2010). *T. gondii* stage conversion is often considered a stress-induced process, as it can be triggered *in vitro* by physical, chemical or nutritional stress, and *in vivo*, by a pro-inflammatory host immune response (Ferreira-da-Silva Mda et al., 2008). However, recent studies have shown that bradyzoite formation can commence very early in infection before induction of a protective immune response (Di Cristina et al., 2008). Moreover, we have recently shown that *T. gondii* spontaneously differentiates to bradyzoites in skeletal muscle cells (SkMCs) that have terminally differentiated to mature, syncytial myotubes, but not in myoblast precursor cells (Ferreira-da-Silva Mda et al., 2009; Swierzy and Luder, 2015), suggesting that the cellular microenvironment plays a decisive role in triggering bradyzoite formation. The possibility that host factors directly initiate stage differentiation may explain the predilection of *T. gondii* to form tissue cysts and to persist in brain and muscle tissues (Luder and Rahman, 2017). Functional analyses revealed that knockdown of the negative cell cycle regulator testis-specific Y-encoded-like protein 2 (Tspyl2) in SkMCs prevents myotube formation and at the same time abolishes *T. gondii* bradyzoite and tissue cyst formation (Swierzy and Luder, 2015). While these findings suggest that stage differentiation might be coordinated with the host cell cycle, the actual host cell signal(s) that triggers parasite differentiation remains elusive. We have recently undertaken a genome-wide transcriptional analysis of different host cell types, which supported the hypothesis that cessation of the host cell cycle may sustain bradyzoite formation in SkMCs and neurons (Swierzy et al., 2017). These analyses also highlighted potential links between parasite differentiation and distinct transcriptomic signatures of genes related to glucose metabolism and the pentose phosphate pathway (PPP), the cell redox homeostasis, and protein catabolism (Swierzy et al., 2017). Conversely, a switch towards a glycolytic metabolism in host cells has been shown to favor tachyzoite growth in distinct cell types (Weilhammer et al., 2012).

Herein, we have exploited the ability of C2C12 SkMCs to induce parasite stage conversion in their cell-cycle arrested but not in their proliferating form to identify factors in the host microenvironment that trigger bradyzoite formation. Using transcriptome profiling and stable isotope metabolic labeling we find that redirection of glucose fluxes into either the oxidative branch of the pentose phosphate pathway (OxPPP) or the tricarboxylic acid (TCA) cycle associates with repression or promotion of bradyzoite differentiation, respectively. We also show that ROS levels and the host redox balance may provide the critical signals that restrain or activate parasite differentiation. We propose a metabolic mechanism that links the cell cycle and proliferation of the host cell to the formation of *T. gondii* tissue cysts.

## Results

### Divergent Transcriptomes of Myoblasts and Myotubes Before and After Infection With *T. gondii*

We have previously shown that *T. gondii* tissue cyst formation occurs spontaneously in differentiated SkMC myotubes but not in their proliferating myoblast precursors (Swierzy and Luder, 2015). In order to identify host cell processes that trigger bradyzoite conversion in SkMCs, we compared the transcriptomes of C2C12 myoblasts and *in vitro*-differentiated myosin heavy chain-positive C2C12 myotubes (**Figure S1A**) before and after 24 hours of infection with *T. gondii* by high-throughput RNA sequencing. Intracellular *T. gondii* parasites were readily detected in both myoblasts and myotubes, with similar levels of infection in both cell types (**Figure S1A**). As expected, RT-qPCR revealed higher levels of the early marker bradyzoite antigen 1 (BAG1) mRNA in parasites within myotubes as compared to those in myoblasts (**Figure S1B**) (Swierzy and Luder, 2015).

**Figure 1:**
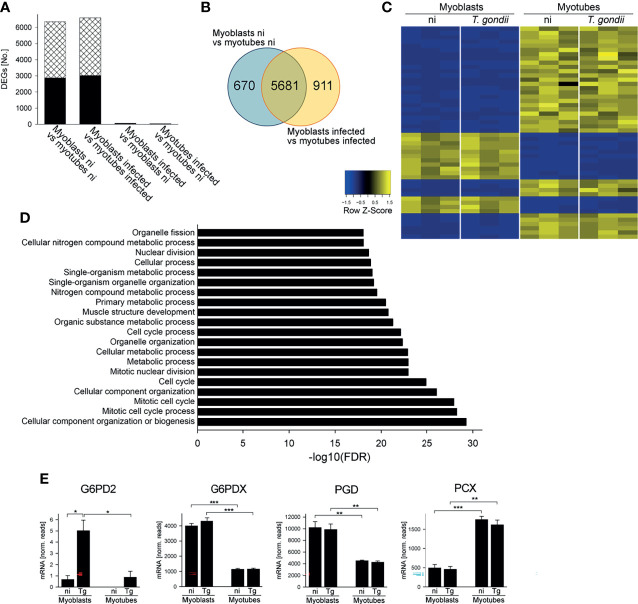
Myoblasts and myotubes during infection with *T. gondii* diverge in their transcriptome profiles including multiple mRNAs of carbohydrate metabolic enzymes. C2C12 myoblasts and *in vitro*-differentiated C2C12 myotubes were infected with *T. gondii* at a MOI of 5:1 for 24 hours or were left non-infected. Total RNA from three biological replicates each was used to prepare cDNA libraries which were then sequenced using Illumina technology. Reads were mapped to the *Mus musculus* reference genome. **(A)** Numbers of differentially expressed genes (DEGs; *p_adjusted_* < 0.05) which were up- or down-regulated (black bars and cross-hatched bars, respectively) between myoblasts and myotubes or after infection as indicated. **(B)** Venn diagram of total DEGs identified between myoblasts and myotubes that were either non-infected (ni) or *T. gondii*-infected. **(C)** The top 50 genes that were most significantly regulated between infected myoblasts and myotubes were hierarchically clustered. Their expression levels from three biological replicates each of non-infected (ni) and infected (*T. gondii*) myoblasts and myotubes were visualized in a heatmap. **(D)** DEGs identified between infected myoblasts and myotubes were functionally analyzed for enrichment of gene ontology (GO) terms. The 20 GO biological processes which were most significantly enriched are depicted. **(E)** Expression levels of G6PD2, G6PDX, PGD and PCX were extracted from the RNAseq data sets and are depicted as means ± S.E.M. from three biological replicates. Significant differences were identified by Student’s *t*-test (*p < 0.05; **p < 0.01; ***p < 0.001).

RNAseq of cDNA libraries of three independent mRNA samples showed that a similar number of genes were differentially regulated between myoblasts and myotubes regardless of whether they were infected with *T. gondii* for 24 hours (6592 genes) or not (6351 genes) (DEGs; FDR-corrected *p* < 0.05) (**Figure 1A**). Between 2872 and 3013 of the DEGs were significantly up-regulated and between 3479 and 3579 of DEGs down-regulated in infected and uninfected myoblasts and myotubes, respectively. Infection with *T. gondii* for 24 hours had a minor impact on the transcriptomes of host cells with only 70 and 38 genes being up-regulated in myoblasts or myotubes, respectively, and none being down-regulated (**Figure 1A**). This is a substantial lower number of parasite-induced DEGs than we identified in a previous study in myotubes (Swierzy et al., 2017) that is likely due to different preparations of RNAseq samples and/or different algorithms to analyze data. It is interesting to note however, that out of the 7262 total DEGs identified in this study between myoblasts and myotubes, 5681 overlapped irrespective of infection, whereas 670 and 911 were specifically regulated before or after parasite infection, respectively (**Figure 1B**). This indicated a significant number of genes that were differentially regulated between myoblasts and myotubes in a *T. gondii*-dependent manner. A heat-map of the 50 most significantly regulated genes between infected myoblasts and myotubes and a cluster dendrogram validated the heterogeneous expression profiles of both host cell types and the minor impact of the parasite infection (**Figures 1C** and **S2**). Similar results were obtained when comparing non-infected myoblasts and myotubes (**Figure S3A**). Functional analysis revealed that among the DEGs regulated between *T. gondii*-infected myoblasts and myotubes, gene ontology (GO) terms related to the cell cycle, mitosis, organelle division and muscle structure development were most prominently enriched, as expected (**Figure 1D**). Biological processes related to the cell cycle and muscle development appeared to differ also between non-infected myoblasts and myotubes (**Figure S3B**). Similarly, multiple genes related to metabolic processes, including primary carbon and nitrogen metabolism were also differentially regulated in infected and non-infected myotubes and myoblasts, including a number of pathways that we have previously proposed to be involved in regulating *T. gondii* differentiation (Swierzy et al., 2017).

### Enzymes of the Central Carbohydrate Metabolism Are Differently Expressed in Myoblasts and Myotubes During *T. gondii* Infection

Transcripts encoding enzymes involved in glycolysis, the TCA cycle and associated anaplerotic reactions, the OxPPP and glycogen metabolism were differentially expressed in *T. gondii*-infected myoblasts and myotubes (**Table S1**). In particular, mRNA levels for glucose-6-phosphate dehydrogenase 2 (G6PD2), glucose-6-phosphate dehydrogenase X-linked (G6PDX) and 6-phosphogluconate dehydrogenase (PGD) were all higher in myoblasts as compared to myotubes (**Figure 1E**), consistent with our previous data suggesting that the PPP may be a putative host cell regulator of *T. gondii* stage differentiation (Swierzy et al., 2017). G6PD2 mRNA was further elevated in myoblasts after *T. gondii* infection (**Figure 1E**) whereas G6PDX and PGD mRNAs were not regulated in response to the parasite. RT-qPCR validated the increased expression of G6PD2, G6PDX and PGD mRNAs in myoblasts as compared to myotubes (*p* < 0.05, ANOVA; **Figure S4A–C**). Furthermore, the kinetics of mRNA levels indicated higher expression of G6PD2, G6PDX and PGD in myoblasts particularly at 4 hours p.i. and a decline until 24 hours of infection. They remained however elevated in myoblasts as compared to myotubes until 48 hours p.i. (**Figures S4A–C**). Conversely, mRNA of pyruvate carboxylase (PCX), which catalyzes the anaplerotic/gluconeogenic interconversion of oxaloacetate and pyruvate, was significantly elevated in myotubes as compared to myoblasts irrespective of *T. gondii* infection (**Figures 1E**, **S4D**). PCX mRNA was already higher in myotubes than in myoblasts at 4 hours p.i. and further diverged until 48 hours between both cell types (**Figure S4D**). Furthermore, *T. gondii*-infected myotubes expressed higher mRNA levels of several enzymes of glycolysis and glycogen metabolism as compared to myoblasts (**Table S1**). Collectively, these results suggested that the differentiation of myoblasts to myotubes may be associated with substantial rewiring of central carbon metabolism that is only modestly affected by *T. gondii* infection.

### Differential Catabolism of Glucose in the PPP and the TCA Cycle in *T. gondii*-Infected Myoblasts and Myotubes

**Figure 2:**
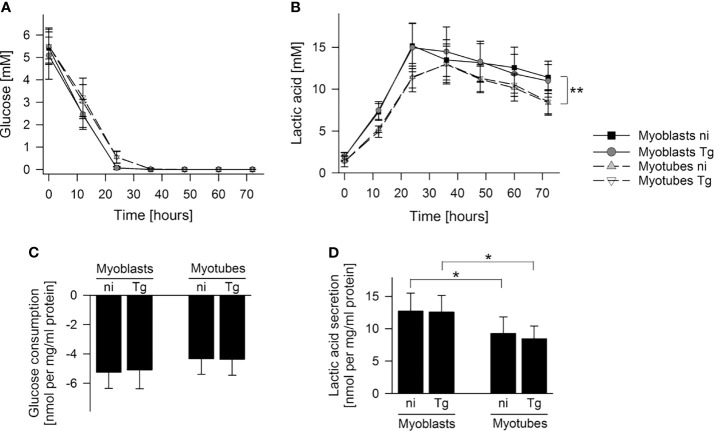
Myoblasts maintain a higher lactic acid secretion rate than myotubes. C2C12 myoblasts and *in vitro*-differentiated C2C12 myotubes were infected with *T. gondii* or were left non-infected. Cell culture supernatants were collected at time points as indicated and metabolites were profiled by GC-MS. **(A, B)** Glucose and lactic acid concentrations were determined in cell culture supernatants of non-infected (ni) and *T. gondii-*infected (Tg) myoblasts and myotubes between 0 and 72 hours of infection. Data are means ± S.E.M. from three independent experiments; significant differences were identified by ANOVA (**p < 0.01). **(C, D)** Glucose consumption and lactic acid secretion were calculated during the initial 24 hours of infection and were normalized to total protein. Results are means ± S.E.M. from three independent experiments. Significant differences were identified by Student’s *t*-test (*p < 0.05).

To further investigate global changes in metabolism of infected and uninfected myoblasts and myotubes, we measured rates of glucose utilization and lactate production in the culture supernatants by gas chromatography-mass spectrometry (GC-MS). Both cell types rapidly consumed glucose, with production of lactate, indicating that they have a strongly glycolytic metabolism. However, myoblasts appeared to deplete extracellular glucose at a faster rate (24 hours *versus* 36 hours) and to consume more glucose during the first 24 hours than myotubes, although this was not statistically different (**Figures 2A, C**). They were found to also produce more lactate (*p* < 0.05; Student’s *t*-test) at a faster rate (*p* < 0.01; ANOVA) than myotubes, indicating that myoblasts have an appreciably higher rate of glycolysis (**Figures 2A, B, D**). The uptake of glucose and production of lactate was not significantly affected by their infection status (**Figures 2C, D**). Interestingly, both cells switched to using lactate after depletion of glucose, which may reflect the uptake and catabolism of this carbon source in the mitochondria.

**Figure 3:**
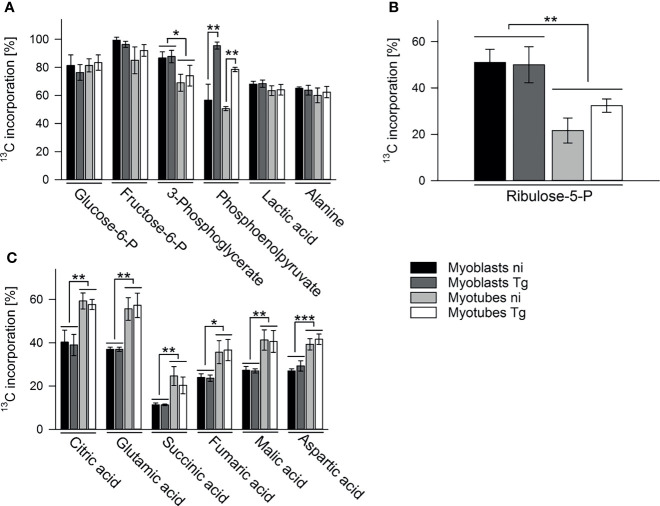
Myoblasts and myotubes during infection with *T. gondii* differentially catabolize glucose in the PPP and the TCA cycle. C2C12 myoblasts and *in vitro*-differentiated myotubes were infected with *T. gondii* for 6 hours or were left non-infected and were then incubated in complete medium containing ^13^C-U-glucose for 4 hours. ^13^C enrichment into intermediates in glycolysis **(A)**, the oxPPP **(B)** and the TCA cycle **(C)** was determined by GC-MS. Data are means ± S.E.M. from three independent experiments; significant differences were identified by ANOVA (*p < 0.05; **p < 0.01; ***p < 0.001).

To further investigate the metabolic changes associated with myoblast to myotube differentiation and/or parasite infection, infected or uninfected myotubes and myoblasts were metabolically labeled with ^13^C-glucose (6 hours p.i. in the case of infected cells) for 4 hours and measurement of ^13^C-enrichments in intracellular metabolites determined by GC-MS. As expected, intracellular pools of glycolytic intermediates were efficiently labeled (55% to 95%) in both infected and uninfected myotubes and myoblasts (**Figures 3A**, **S5A**). The level of labeling (and size of steady state pools; **Figure S5A**) of individual glycolytic intermediates in each host cell type was generally unaffected by the infection, with the exception of the glycolytic intermediate, phosphoenolpyruvate (PEP), which was highly elevated in both infected myotubes and myoblasts (**Figure 3A**). The high level of ^13^C-enrichment in PEP in infected cells compared to other glycolytic intermediates may reflect the uptake of ^13^C-glucose by the intracellular tachyzoites and parallel labeling of glycolytic intermediates.

**Figure 4:**
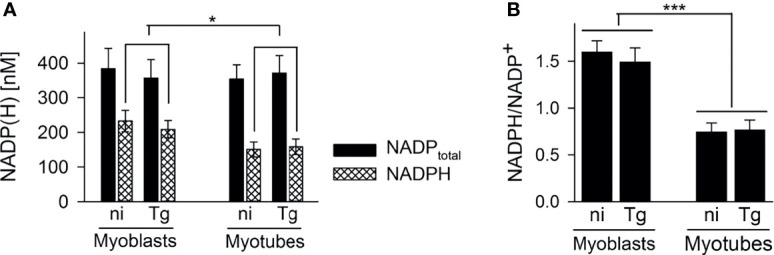
Myoblasts contain more NADPH during infection with *T. gondii*. C2C12 myoblasts and *in vitro*-differentiated myotubes were infected with *T. gondii* or were left non-infected. After cell extraction at 48 hours of infection, total NADP (i.e. NADP^+^ and NADPH) and NADPH levels were determined using a colorimetric assay and standards of known concentrations. Bars in **(A)** represent mean concentrations ± S.E.M. of total NADP (black bars) and NADPH (cross-hatched bars) from non-infected (ni) and *T. gondii*-infected (Tg) cells as indicated. Bars in **(B)** represent the mean ratios ± S.E.M. of NADPH to NADP^+^ (i.e. total NADP minus NADPH) from four independent experiments. Significant differences were identified by ANOVA (*p < 0.05; ***p < 0.001).

^13^C-glucose was also incorporated into intermediates in the OxPPP and the TCA cycle in both cell types (**Figures 3B, C**). Total abundances of OxPPP and TCA cycle intermediates did not differ between cell types or after infection (**Figures S5B, S5C**). However, levels of ^13^C-enrichment in OxPPP intermediates were consistently higher in myoblasts compared to myotubes indicating elevated OxPPP flux in myoblasts (*p* < 0.01; **Figure 3B**). Enhanced flux into the OxPPP in myoblasts was further supported by the finding that levels of NADPH were significantly elevated in these cells, compared to myotubes (**Figure 4A**, *p* < 0.05; ANOVA). The OxPPP plays an important role in regulating the redox balance of cells, by regenerating cytoplasmic pools of NADPH, particularly under conditions of oxidative stress (Stincone et al., 2015). NADPH/NADP^+^ ratios in myoblasts were approximately twice as high as those in myotubes (**Figure 4B**; *p* < 0.001; ANOVA) supporting the notion that the OxPPP plays a key role in regulating redox balance in myoblasts.

**Figure 5:**
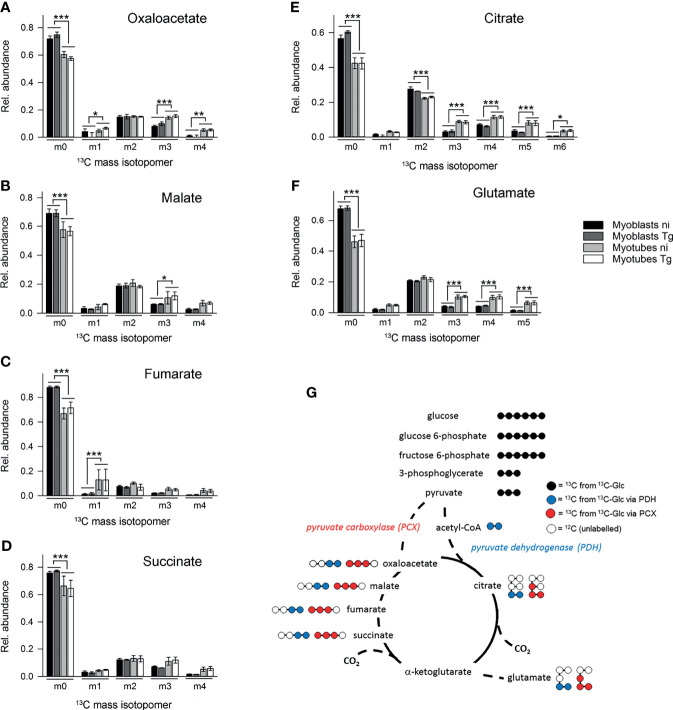
Myotubes during infection with *T. gondii* channel more glycolytic glucose *via* the pyruvate carboxylase into the TCA cycle than myoblasts. C2C12 myoblasts and *in vitro*-differentiated myotubes were infected with *T. gondii* for 6 hours or were left non-infected and were then incubated in complete medium containing ^13^C-U-glucose for 4 hours. **(A–F)** The molar ratio of mass isotopologues (m0-m6) of different TCA cycle intermediates and glutamate are indicated. Bars represent means ± S.E.M. from three independent experiments; significant differences were identified by ANOVA (*p < 0.05; **p < 0.01; ***p < 0.001). **(G)** Schematic of the number of ^13^C atoms in metabolites derived from ^13^C-U-glucose catabolized through glycolysis (black) and channeled into the TCA cycle either *via* the PCX (red) or the PDH (blue).

In contrast, ^13^C-enrichment in TCA cycle intermediates was consistently elevated in myotubes, compared to myoblasts, indicating increased flux of pyruvate into mitochondrial metabolism and oxidative phosphorylation in these cells (**Figure 3C**). Glycolytic intermediates can enter the TCA cycle as acetyl-CoA (two carbon units) produced by pyruvate dehydrogenase (PDH) or as oxaloacetic acid (four carbon unit), generated by carboxylation of pyruvate by pyruvate carboxylase (PCX). The rate at which pyruvate enters the TCA cycle *via* PDH or anaplerotic reactions can be assessed from the relative abundance of +2 (and +4, PDH), +3 (PCX) or +5 (both pathways) mass isotopologues of TCA cycle intermediates in ^13^C-U-glucose-labeled cells (**Figure 5G**). ^13^C-glucose labeling of myotubes led to increased enrichment of citrate and α-ketoglutarate (represented by glutamate) with +3, +4 and +5 isotopologues compared to myoblasts, indicating higher flux of pyruvate into this cycle *via* both PDH and anaplerotic pathways (**Figures 5E, F**). Similar enrichment for +3 and +4 isotopologues in malate and oxaloacetate supported these conclusions (**Figures 5A, B**). The presence of fully labeled citrate (+6) in myotubes, but not myoblasts, provided further support for complete oxidation of glucose in myotubes. In contrast, all of the TCA cycle intermediates in myoblasts were predominantly labeled with two ^13^C-atoms, indicating that pyruvate primarily enters the TCA cycle *via* PDH and is oxidized through only one cycle (**Figures 5A–F**). Collectively, these data indicate that increased expression of OxPPP enzymes in myoblasts and of PCX in myotubes is associated with concomitant changes in respective metabolic fluxes in both cell types.

### *T. gondii*-Infected Myoblasts and Myotubes Differ in Redox Homeostasis

**Figure 6:**
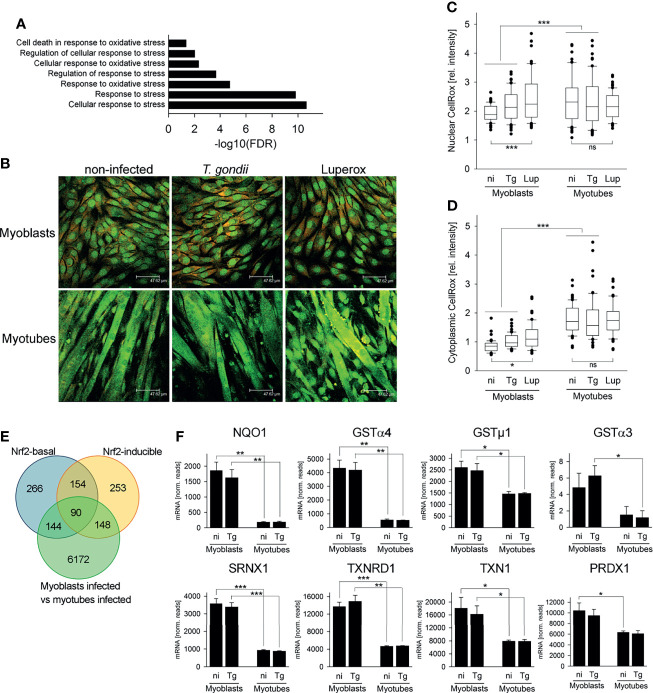
The cellular redox homeostasis and Nrf2-dependent stress responses differ between myoblasts and myotubes during infection with *T. gondii*. C2C12 myoblasts and *in vitro*-differentiated myotubes were infected with *T. gondii* for 24 or 48 hours or were left non-infected. **(A)** RNA isolated at 24 hours after infection or from non-infected controls was used for high-throughput mRNA sequencing as described in Figure legend 1. DEGs identified between infected myoblasts and myotubes were analyzed for significantly enriched GO terms which are related to the stress response. Results are from three biological replicates. **(B–D)** At 48 hours after infection, ROS were labelled in live cells using a fluorogenic CellROX™ oxidative stress probe (green). Note that the CellROX™ Green reagent upon oxidation binds to DNA, and the signal is therefore primarily localized in the nucleus and in mitochondria. After fixation, cells were additionally labelled by propidium iodide (red). As a positive control, non-infected myoblasts and myotubes were partially treated with tert-butyl peroxide (Luperox) for 1 hour before ROS staining. **(B)** Representative images were recorded by confocal laser scanning microscopy. **(C, D)** CellROX™ Green intensities from nuclear **(C)** and cytoplasmic **(D)** regions were recorded from confocal laser scanning microscopic images and were normalized to PI intensities from the same regions. Data are from 60 individual cells each of non-infected (ni), *T. gondii*-infected (Tg) or Luperox (Lup)-treated cells from 3 independent experiments. Significant differences were identified by ANOVA (*p < 0.05; ***p < 0.001; ns, not significant). **(E)** Constitutive and inducible direct target genes of the stress-related transcription factor Nrf2 [Nrf2-basal or Nrf2-inducible, respectively (Malhotra et al., 2010)] were compared with DEGs between *T. gondii*-infected myoblasts and myotubes as identified by RNAseq described in Figure legend 1. **(F)** Expression levels of NAD(P)H dehydrogenase (quinone 1; NQO1), glutathione S-transferases (GSTs) α4, µ1 and α3, sulfiredoxin 1 homolog (SRNX1), thioredoxin reductase 1 (TXNRD1), thioredoxin 1 (TXN1) and peroxiredoxin 1 (PRDXN1) were extracted from the RNAseq data sets and are depicted as means ± S.E.M. from three biological replicates. Significant differences were identified by Student’s *t*-test (*p < 0.05; **p < 0.01; ***p < 0.001).

We next investigated how decreased flux into the OxPPP in SkMCs may induce bradyzoite formation. Our RNAseq data sets revealed a significant enrichment of genes involved in stress responses and, more specifically, in oxidative stress responses among the DEGs identified between *T. gondii*-infected myoblasts and myotubes (**Figure 6A**). Differences in oxidative stress in myoblasts and myotubes would also be consistent with the observed differences in NADHP/NADP^+^ ratios in these cells (see **Figure 4**). We therefore investigated whether reactive oxygen species (ROS) levels were elevated in myotubes compared to myoblasts by CellROX™ Green labelling. CellROX™ Green is a fluorogenic probe that, when oxidized, binds to DNA and thus primarily localizes to the nucleus and to mitochondria. In myoblasts, CellROX™ Green predominantly localized to the nuclei, whereas in myotubes it predominantly stained the cytoplasm (**Figure 6B**). The strong labelling of the cytoplasmic compartment of myotubes likely indicates binding of the oxidized probe to mitochondrial DNA, as the number of mitochondria increase during myogenesis (Remels et al., 2010; Malinska et al., 2012). Quantitation of fluorescence intensities by confocal laser scanning microscopy revealed however also significantly higher CellROX Green™ signals when normalized to propidium iodide in the cytoplasm of both non-infected and *T. gondii*-infected myotubes than in myoblasts (**Figure 6D**; *p* < 0.001; ANOVA). Moreover, the relative CellROX™ intensities were also higher in the nuclei of myotubes than in those of myoblasts, irrespective of infection (**Figure 6C**; *p* < 0.001). These results indicated that ROS levels are substantially higher in parasite-infected myotubes as compared to myoblasts, as previously reported (Piao et al., 2005; Malinska et al., 2012). Treatment of cells with the oxidant tert-butyl peroxide (Luperox) augmented the relative CellROX™ Green intensities in the nuclei and the cytoplasm of myoblasts (*p* < 0.001 and *p* < 0.05, respectively) but not in myotubes (**Figures 6C, D**). The latter may be due to the overall high CellROX™ Green signals after differentiation to myotubes.

The cellular response to stress including oxidative stress is largely governed by binding of the transcription factor Nuclear factor E2 p45-related factor 2 (Nfe2l2, referred to as Nrf2) to the promoters of a large number of antioxidant and survival genes (Hirotsu et al., 2012; Suzuki and Yamamoto, 2017). We therefore analyzed DEGs between *T. gondii*-infected myoblasts and myotubes for the presence of direct Nrf2 target genes as previously identified by genome-wide ChIPseq and RNAseq (Malhotra et al., 2010). Remarkably, out of the 654 constitutive (Nrf2-basal) and the 645 inducible Nrf2 targets, 234 (35.8%) and 238 (36.9%) genes, respectively, were differentially expressed in myoblasts and myotubes during infection with *T. gondii* (**Figure 6E** and **Table S2**). Direct Nrf2 target genes were both up- and down-regulated in both cell types, but particularly among the inducible Nrf2 targets, more genes were up-regulated in myoblasts than down-regulated (80 out of 148; **Table S2**). Furthermore, several *bona fide* Nrf2 target genes including detoxifying NAD(P)H dehydrogenase (quinone 1) and glutathione S-transferases (GSTs) α3, α4 and µ1, as well as the antioxidants sulfiredoxin 1 homolog, thioredoxin reductase 1, thioredoxin 1 and peroxiredoxin 1 were expressed at higher levels in myoblasts than in myotubes, irrespective of infection (**Figure 6F** and **Table S2**). Remarkably, G6PDX, PGD and PCX are also direct targets of Nrf2 (Malhotra et al., 2010), and their increased (G6PDX and PGD) or decreased (PCX) expression in myoblasts as compared to myotubes (see **Figure 1E**) also indicated differential activities of the Nrf2 pathway in both cell types. Finally, Nrf2 alleviates inflammation and suppresses pro-inflammatory IL-6 and IL-1β, at least in macrophages (Kobayashi et al., 2016), and mRNAs of these genes were indeed lower in infected myoblasts as compared to myotubes (IL-6: 0.13-fold; IL-1β: 0.028-fold). Together, these results suggest that Nrf2 activity is increased in *T. gondii*-infected myoblasts thereby diminishing oxidative stress in these cells.

### ROS Regulate *T. gondii* Bradyzoite Formation in SkMCs

**Figure 7:**
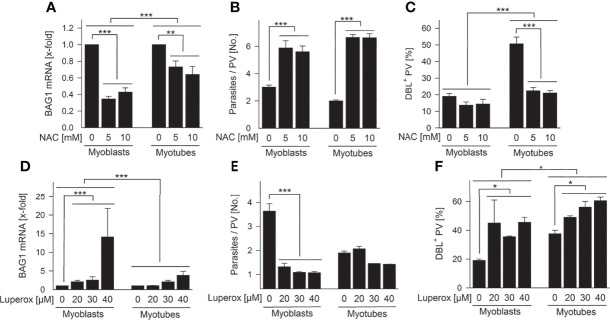
Intracellular ROS regulate *T. gondii* bradyzoite formation in SkMCs. C2C12 myoblasts and *in vitro*-differentiated myotubes were treated with N-acetyl cysteine (NAC; **A–C**) or tert-butyl peroxide (Luperox; **D–F**) or were mock-treated as indicated. One hour later, they were infected with *T. gondii*. **(A, D)** After isolation of total RNA at 48 hours of infection and after reverse transcription, *T. gondii* BAG1 and *T. gondii* actin mRNAs were quantitated by real-time PCR. Bars indicate the treatment-induced regulation of BAG1 mRNA as compared to mock-treated controls and normalized to actin mRNA (means ± S.E.M., n ≥ 3). **(B, E)** Alternatively, cells were fixed at 48 hours after infection, and parasites and SkMCs fluorescently labelled using *T. gondii*-specific antibodies and propidium iodide, respectively. The average number of parasites per parasitophorous vacuole (PV) was determined from 100 PVs; results indicate means ± S.E.M. (n ≥ 2). **(C, F)** After cells were fixed at 72 hours post infection, *T. gondii* tissue cysts were labelled with *Dolichos biflorus* lectin (DBL), total parasites were labelled using a *T. gondii*-specific antiserum, and host cells were visualized using propidium iodide. The percentage of DBL-positive tissue cysts was determined in 100 *T. gondii* PVs; results indicate means ± S.E.M. (n ≥ 2). **(A–F)** Significant differences were identified by ANOVA (*p < 0.05; **p < 0.01; ***p < 0.001).

To determine the impact of ROS on *T. gondii* stage differentiation, we modulated ROS in SkMCs and evaluated BAG1 expression, parasite proliferation and tissue cyst formation. Treatment of infected myoblasts and myotubes with 5 – 10 mM of the antioxidant, N-acetyl cysteine (NAC) significantly down-regulated *T. gondii* BAG1 mRNA levels in both host cell types (**Figure 7A**; *p* < 0.01 for myotubes, *p* < 0.001 for myoblasts; ANOVA). BAG1 mRNA decreased more extensively in parasites residing in myoblasts than in those in myotubes (*p* < 0.001; ANOVA). The transition from tachyzoites to bradyzoites is regularly accompanied by reduced parasite division (Bohne et al., 1994), and mature bradyzoites enter a G_0_ non-replicative cell cycle stage (Radke et al., 2003). Specifically, proliferation of the parasite significantly increased in both cell types treated with NAC (**Figure 7B**; *p* < 0.001; ANOVA). Finally, NAC strongly decreased formation of the characteristic CST1-rich cyst wall (Tomita et al., 2013) by *T. gondii* in myotubes (*p* < 0.001; ANOVA) as indicated by labelling with the *Dolichos biflorus* lectin at 72 hours after infection (**Figure 7C**). The oxidant Luperox increased BAG1 mRNA particularly in parasites within myoblasts (*p* < 0.001; ANOVA) and only to a small extent in those in myotubes (**Figure 7D**). Thus, parasites within both cell types again differed significantly in their BAG1 mRNA response to redox regulation in myoblasts and myotubes. The replication of *T. gondii* decreased accordingly after treatment with Luperox, with a clearly stronger inhibition in myoblasts than in myotubes (**Figure 7E**; *p* < 0.001; ANOVA). Finally, tissue cyst formation by *T. gondii* was augmented after treatment of both cell types, but increased more strongly in myoblasts than in myotubes (**Figure 7F**). Collectively, these data clearly indicate that increased ROS levels trigger *T. gondii* bradyzoite differentiation in SkMCs and that host cell specific differences in intracellular ROS levels largely contribute to different levels of spontaneous differentiation in myoblasts, compared to myotubes.

## Discussion

There is increasing evidence that the host cell environment plays an important role in regulating *T. gondii* tachyzoite-to-bradyzoite differentiation and bradyzoite tropism [reviewed in (Luder and Rahman, 2017)]. Here, we have shown that changes in the metabolism of SkMCs may regulate non-immune induced bradyzoite differentiation (Ferreira-da-Silva Mda et al., 2009; Swierzy and Luder, 2015). Specifically, we show that high flux through the OxPPP and elevated NADPH/NADP^+^ ratios is associated with tachyzoite proliferation in myoblasts. Since *T. gondii* bradyzoite differentiation requires a growth shift towards slower cell cycle progression (Radke et al., 2003), tachyzoite replication in myoblasts may repress bradyzoite-specific genes through distinct ApiAP2 transcription factors (Radke et al., 2018). Conversely, increased ROS production in myotubes, possibly by enhanced mitochondrial metabolism, promotes bradyzoite formation in these cells. Thus, changes in host cell metabolism may exert both positive and negative effects on stage differentiation of *T. gondii*.

Myoblasts and myotubes exhibit marked differences in growth rate (Walsh and Perlman, 1997) which may underlie the significant metabolic difference between the two cell types (DeBerardinis et al., 2008; Aguilar and Fajas, 2010). While both cell types utilize glucose, our genome-wide transcriptomic analysis and stable isotope labeling studies, indicate that flux of glucose-6-phosphate into the OxPPP is significantly elevated in rapidly dividing myoblasts. This may reflect high demands of myoblasts for ribose-5-phosphate for nucleotide biosynthesis as well as NADPH for anabolic purposes and redox balance (Stincone et al., 2015). In contrast, non-dividing myotubes exhibit lower rates of glycolysis (as shown by lactate production) and increased flux of pyruvate into the TCA cycle *via* either the PDH or anaplerotic (i.e. PCX) reactions. These metabolic transitions are similar to those that occur in other animal cell types (i.e. lymphocytes, macrophages and tumor cells) that alternate between different growth states (Warburg, 1956; Wang et al., 1976; Aguilar and Fajas, 2010).

It remains to be resolved how exactly the OxPPP sustains the rapidly proliferating tachyzoite stage. Although we provide evidence that formation of NADPH may be involved through counteracting oxidative stress in myoblasts (see below), it is possible that ribose sugars generated by the host OxPPP or their derivatives could be scavenged by intracellular parasites and facilitate tachyzoite growth. Similarly, *T. gondii* is auxotrophic for other key metabolic building blocks, including purines, several amino acids, polyamines, cholesterol, choline and most vitamins (Chaudhary and Roos, 2005; Fox et al., 2007; Lüder and Seeber, 2016). Whether and to what extent the intracellular levels of these metabolites regulate parasite growth remains to be determined.

Our transcriptomic analysis suggested differences in oxidative stress in myotubes *versus* myoblasts leading to increased expression of several *bona fide* Nrf2 anti-oxidant and detoxification genes in *T. gondii*-infected myoblasts as compared to myotubes. Nrf2 is a master regulator of the transcriptional response to oxidative and other environmental stresses by binding to *cis*-regulatory antioxidant response element (ARE) DNA recognition sites (Malhotra et al., 2010; Suzuki and Yamamoto, 2017). Different expression levels of anti-oxidants in myoblasts and myotubes is in line with our previous finding that genes involved in redox homeostasis are differentially expressed in SkMCs and neurons as compared to fibroblasts and astrocytes, i.e. cell types which differ in their ability to sustain *T. gondii* bradyzoite formation (Swierzy et al., 2017). Here, we additionally provide direct evidence that ROS levels are higher in myotubes than in myoblasts. Mitochondria are a major source of ROS in SkMCs (Malinska et al., 2012), and the higher ROS levels in myotubes may derive from the increased amount of mitochondria present after differentiation of SkMCs (Remels et al., 2010; Malinska et al., 2012). In addition, myoblasts are obviously able to detoxify ROS efficiently as suggested by high NADPH/NADP^+^ ratios and increased mRNA levels of various detoxifying enzymes and anti-oxidants in these cells. Importantly, by modulating ROS levels through inhibitors and oxidant treatment we were able to abolish or to promote bradyzoite formation in *T. gondii*, respectively. ROS, particularly non-radical species such as hydrogen peroxide or singlet molecular oxygen at physiological levels, i.e. oxidative eustress, mediate various cellular processes through redox signaling (Sies et al., 2017) and may be associated with calcium-signaling, parasite egress and replication of tachyzoites in fibroblasts (Alves et al., 2021). We suggest that oxidative stress in myotubes may trigger bradyzoite formation, presumably through phosphorylation of the *T. gondii* homologue of the eukaryotic initiation factor 2 α subunit (TgIF2α) by the kinase TgIF2K-B and translational control of ApiAP2 transcription factors (Narasimhan et al., 2008; Augusto et al., 2021). Parasites deficient in TgIF2K-B are indeed unable to properly responding to oxidative stress and to efficiently forming tissue cysts (Augusto et al., 2021).

Nrf2 also regulates various other cellular functions including glucose metabolism (Hirotsu et al., 2012), cell cycle progression and proliferation (Malhotra et al., 2010), and anti-inflammation (Kobayashi et al., 2016). The differential regulation of these functions between myotubes and myoblasts during parasite infection as shown herein or as reported previously (Swierzy et al., 2014; Swierzy and Luder, 2015) all points towards a higher Nrf2 transcriptional activity in infected myoblasts than in myotubes. We therefore propose that Nrf2 activity in infected myoblasts increases the expression of enzymes in the OxPPP as well as anti-oxidant proteins, which in turn decrease ROS and potentially provide anabolic precursors for sustained tachyzoite replication. On the other hand, the decreased Nrf2 activity in differentiated myotubes does not suffice to efficiently counteract ROS which, after diffusion to the parasite, trigger bradyzoite formation. We also propose that ceasing host cell cycle progression and expression of the cell cycle inhibitor Tspyl2 as observed in differentiated myotubes (Swierzy and Luder, 2015) promotes *T. gondii* stage differentiation indirectly *via* regulation of the PPP and redox homeostasis rather than by a direct mechanism.

The identification of host cell factors which regulate bradyzoite formation in *T. gondii* significantly furthers our understanding of a critical step in the developmental biology of an intracellular parasite. It may also help to unravel host-to-parasite signaling pathways that explain the predilection of *T. gondii* to form tissue cysts in brain and muscle tissues *in vivo*.

## Materials and Methods

### Culture and Differentiation of Skeletal Muscle Cells (SkMC)

The mouse myoblast cell line C2C12 (European Collection of Animal Cell Cultures (ECACC), Salisbury, UK) was propagated in DMEM supplemented with 10% FCS, 100 U/ml penicillin and 100 µg/ml streptomycin (all reagents from Biochrom, Berlin, Germany). Myoblasts were differentiated to polynucleated and cell cycle-arrested myotubes as described (Takacs et al., 2012). Briefly, at 24 hours after seeding of myoblasts, myogenic differentiation was induced by incubation in DMEM, 2% horse serum (Biochrom) and antibiotics as above. After 6 – 7 days of differentiation and one day before infection, the medium was replaced by DMEM, 10% FCS and antibiotics as above. In parallel, proliferating myoblasts were seeded in the same medium. For metabolome analyses, myotubes and myoblasts were cultured in glucose-free DMEM supplemented with 1 g/L glucose, 10% FCS and antibiotics. In some experiments, myoblasts and myotubes were treated with 5 – 10 mM N-acetyl cysteine (NAC), 20 – 40 µM tert-butyl peroxide (Luperox) or with vehicle (mock), starting at 1 hour prior to infection (all reagents from Sigma-Aldrich, Taufkirchen, Germany).

### Parasite Infection

Tachyzoites of the *T. gondii* type II strain NTE (Gross et al., 1991) were propagated in murine L929 fibroblasts in RPMI 1640 medium supplemented with 1% FCS, 100 U/ml penicillin and 100 µg/ml streptomycin. For infection of SkMCs, freshly egressed parasites were isolated by differential centrifugation as described (Vutova et al., 2007). Briefly, contaminating host cells were pelleted by centrifugation at 34 x *g* for 5 minutes, and parasites were subsequently collected from the supernatant by centrifugation at 1,300 x *g* for 10 minutes. After having been washed extensively, parasites were added to SkMCs at a multiplicity of infection (MOI) of 3.5:1 unless stated otherwise. In order to take cell fusion during myotube formation into consideration, we calculated MOI with regard to myoblast cultures and added the same amount of parasites to myotubes.

### High Throughput RNA Sequencing

At 24 hours after infection, total RNA was isolated from infected and non-infected myoblasts and myotubes using the GenElute Total RNA Miniprep Kit (Sigma-Aldrich, Taufkirchen, Germany) according to the manufacturer instructions. RNA quality was assessed by measuring the RIN (RNA Integrity Number) using an Agilent 2100 Bioanalyzer (Agilent Technologies, Palo Alto, CA). Library preparation for RNA sequencing was performed using the TruSeq™ RNA Sample Prep Kit v2 (Illumina, San Diego, USA; Cat. N°RS-122-2002), starting from 1,000 ng of total RNA. Accurate quantitation of cDNA libraries was performed by using the QuantiFluor™ dsDNA System (Promega, Mannheim, Germany). The size range of the final cDNA libraries (average 350 bp) was determined in DNA 1000 chips using the Bioanalyzer 2100. CDNA libraries were amplified and sequenced by using the cBot and HiSeq2000 from Illumina (single reads of 50 bp; ~30-40 million reads per sample). Sequence images were transformed with Illumina software BaseCaller to bcl files, which were demultiplexed to fastq files with CASAVA v1.8.2. A quality check was done *via* fastqc (v. 0.10.0, Babraham Bioinformatics). Read alignment was performed using STAR v2.3.0 to the mm10 *Mus musculus* reference genome. Data were converted and sorted by samtools 1.2 and reads per gene were counted *via* htseq version 0.6. Data analysis was performed using R (3.2.1) and differential gene expression was analyzed using DESeq2 (v1.8.1). It included normalization of reads applying the ‘median of ratios’ method that accounts for differences in sequencing depth and RNA composition between samples. Candidate genes were filtered to a minimum of a 2-fold change and a FDR-corrected *p* < 0.05 (*p_adjusted_*). For functional analysis, gene ontology enrichment was tested accounting for gene length *via* R-package goseq (v1.20). Sequence data have been deposited in NCBI’s Gene Expression Omnibus and are accessible through GEO Series accession number GSE133952.

### Stable Isotope Labelling, Metabolite Extraction and Analysis by Gas Chromatography-Mass Spectrometry (GC-MS)

Stable isotope labelling experiments were carried out as described previously (Blume et al., 2015) with slight modifications. Briefly, six hours after infection of myoblasts and myotubes with *T. gondii*, the cell culture medium was exchanged with medium in which ^12^C-glucose was replaced by ^13^C-U-glucose (Euriso-top, Saarbrücken, Germany). After incubation for four hours, aliquots of the cell culture media were centrifuged at 14,000 x *g* for 5 minutes, and the supernatants were stored at -80°C. Cells were collected by scraping into ice-cold 80% GC-MS grade methanol supplemented with 1 nmol/sample *scyllo*-inositol as internal standard. They were then sonicated four times 30 seconds each in an ultrasonic bath, and metabolites were extracted in chloroform:methanol:water (1:11:6 v/v) for 20 minutes at 60°C. After removal of insoluble material by centrifugation (10 minutes, 14,000 x *g*, 4°C), phases were separated by addition of water and by sonication. The aqueous phase was dried in a heated Speedvac and stored at -80°C. Polar metabolites were mixed with ice-cold 80% methanol, 1 nmol/sample *scyllo*-inositol, dried, then methoxymated (20 mg/ml methoxyamine in pyridine, overnight) and trimethylsilylated in N,O-bis(trimethylsilyl)trifluoroacetamide with 1% trimethylsilyl (BSTFA-1% TMS, 1 hour, room temperature, reagents from Sigma-Aldrich, Castle Hill, Australia). Apolar metabolites were subjected to methanolysis (0.5 M methanolic HCl at 80°C for 4 hours). Samples were analysed using an Agilent 7890A-5975C GC-MS system with a DB-5MS + DG column (J&W, Agilent, 30m × 0.25 mm, with 10 gap). Chromatograms were processed in MSD Chemstation D.01.02.16 software (Agilent, Santa Clara, USA). The incorporation of ^13^C-atoms was estimated as the percentage of the metabolite pool containing one or more ^13^C-atoms after correction for natural abundance. Total metabolite counts were normalized to *scyllo*-inositol as an internal standard and to the total protein content of each sample as determined by using the Pierce™ BCA assay kit (Thermo Scientific, Schwerte, Germany) according to the manufacturer instructions.

### Metabolite Profiling in Cell Culture Supernatants

For total flux analysis, 10 µl each of cell culture supernatants from infected and non-infected myoblasts and myotubes were removed at the indicated time points. After having been dried in a Speedvac, polar metabolites were methanol-washed, methoxymated and trimethylsilylated, and analysed by GC-MS as described above. Cells were scraped for measuring the total protein content as described above. Metabolite abundance was normalised to total protein content.

### Reverse Transcription and Quantitative PCR

Total RNA was isolated at 4, 24 or 48 hours of infection or from non-infected controls using the GenElute Total RNA Miniprep Kit (see above). Contaminating DNA was digested with amplification grade DNase I as recommended by the manufacturer (Sigma-Aldrich, Taufkirchen, Germany). Thereafter, mRNA from 2 µg of total RNA was reverse transcribed using the Omniscript Reverse Transcription Kit (Qiagen, Hilden, Germany) and oligo(dT) primers. Serial dilutions of cDNA were then amplified in a LightCycler 1.5 using the SYBR Green I FastStart DNA Master^Plus^ Kit (Roche Diagnostics, Mannheim, Germany) and primer pairs as specified in **Table S3**. The relative expression of target genes was determined according to the ΔΔCt method (Pfaffl, 2001) and was normalized to β-actin or else to actin for mouse and *T. gondii* target genes, respectively.

### Fluorescence Staining and Confocal Microscopy

Differentiation of SkMCs, parasite replication and formation of *T. gondii* tissue cysts were evaluated after staining cells with fluorophore-conjugated probes (Swierzy and Luder, 2015). To this end, myoblasts and myotubes grown on glass cover slips were fixed at 24 to 72 hours post infection using 4% paraformaldehyde in 0.1 M sodium cacodylate, pH 7.4. They were then quenched for 10 minutes in 50 mM NH_4_Cl in PBS. Cells were permeabilized and unspecific bindings sites were blocked for 1 hour in 0.1 mg/ml saponin, 1% BSA in PBS. The formation of polynucleated mature myotubes was validated by staining myoblasts and myotubes during 1 hour with 2 µg/ml rabbit anti-myosin heavy chain (MyHC; H-300, Santa Cruz Biotechnology, Heidelberg, Deutschland) diluted in 0.1 mg/ml saponin, 1% BSA in PBS. Alternatively, the cyst wall of *T. gondii* tissue cysts was labelled for 1 hour in 3.125 µg/ml biotin-conjugated *Dolichos biflorus* lectin (DBL; Sigma-Aldrich, Taufkirchen, Germany), and/or the total parasite population was labelled using rabbit or mouse anti-*Toxoplasma* serum (1:2000; diluted as above). After having been washed in 0.1 mg/ml saponin in PBS, primary probes were fluorescently labeled with 4 µg/ml Cy2-conjugated streptavidin and 7.5 µg/ml Cy5-conjugated donkey F(ab’)_2_ anti-rabbit or anti-mouse IgG (H+L), or else with 3.75 µg/ml DyLight488-conjugated donkey F(ab’)_2_ anti-rabbit IgG (H+L) (in case of staining myotubes or the total parasite population only; secondary reagents from Dianova, Hamburg, Germany). After having been washed as above, nuclei acids were stained with 5 µg/ml propidium iodide in PBS for 5 minutes. Cells were then mounted with Mowiol 4-88 (Calbiochem-Novabiochem, Bad Soden, Germany) and analyzed by confocal laser scanning microscopy using a Leica TCS SP2 (Leica Microsystems, Heidelberg, Germany).

### NADP^+^/NADPH Measurement

Cellular levels of NADP^+^ and NADPH were determined using a colorimetric quantification kit according to the manufacturer instructions (Sigma-Aldrich, Taufkirchen, Germany). Briefly, 1 – 1.5 x 10^6^ infected or non-infected SkMCs were extracted for 10 minutes at 4°C in 300 µl of extraction buffer. Insoluble material was pelleted for 10 minutes at 10,000 x *g* and 4°C, and the supernatants filtered through Vivaspin 500 filters with a MWCO of 10 kDa (Sartorius, Göttingen, Germany). NADP^+^ was then decomposed from 100 µl of each sample by incubation at 60°C for 30 minutes. Any NADP^+^ within unknown samples either pretreated to remove NADP^+^ or left untreated was then converted to NADPH by incubation with NADP^+^ cycling enzyme and NADP^+^ cycling buffer for 5 minutes. Thirty minutes after adding developer solution, NADPH was then detected by repeatedly measuring the absorbance at 450 nm in a Victor V multilabel plate reader during 1 hour (PerkinElmer Life Sciences, Rodgau-Jügesheim, Germany). NADPH concentrations (after decomposing NADP^+^) and NADP_total_ (NADP^+^ + NADPH, i.e. without sample heating and after converting NADP^+^ to NADPH) in unknown samples were calculated from a standard curve (0 – 100 picomole of NADPH). All measurements were done in duplicate.

### ROS Labelling

Reactive oxygen species (ROS) were labeled using the CellROX™ Green Reagent according to the manufacturer instructions (Molecular Probes, Eugene, USA). CellROX™ Green predominantly detects hydroxyl radicals and superoxide anions and only to low extent tert-butyl-hydroperoxide and do not detect hydrogen peroxide (Choi et al., 2015) (www.thermofisher.com). Briefly, live infected and non-infected myoblasts and myotubes were stained with 5 µM CellROX™ Green Reagent in complete medium for 30 minutes at 37°C, 5% CO_2_. As positive control, non-infected cells were partially treated with 200 µM of the oxidant tert-butyl peroxide (Luperox) for 60 minutes at 37°C and 5% CO_2_ prior to staining. Cells were then washed in PBS, pH 7.4, and were fixed in 4% paraformaldehyde in 0.1 M sodium cacodylate, pH 7.4 for 15 minutes. After washing, nucleic acids were stained with propidium iodide, and cells were mounted and analyzed by confocal laser scanning microscopy as described above.

### Statistical Analyses

Results are expressed as means ± S.E.M. of at last three independent experiments unless stated otherwise. Significant differences between means of two or more variables were identified by Student’s *t*-test or by multifactorial ANOVA, respectively using Statistica 13 (Dell, Round Rock, USA). *P*-values of less than 0.05 were considered significant.

## Data Availability Statement

The data presented in the study are deposited in the NCBI's Gene Expression Omnibus repository, accession number GSE133952.

## Author Contributions

CL conceived and coordinated the study, and he designed and supervised experiments. MB contributed to study and experiment design. MR, IS, and MB performed the experiments. MM and GS provided technical resources and supervised experiments. BD, MR, IS, MB, and CL analysed data. CL wrote the manuscript draft. CL, MB, and MM finalized the manuscript. All authors contributed to the article and approved the submitted version.

## Funding

This study was supported by the Federal Ministry of Education and Research (BMBF, TOXONET-02 consortium, grant number 01Kl1002B to CL) and a PhD scholarship from Interweave Erasmus Mundus Action 2 Partnership to MR. MM is a NHMRC Principal Research Fellow. The funders had no role in study design, data collection and analysis, decision to publish, or preparation of the manuscript.

## Conflict of Interest

The authors declare that the research was conducted in the absence of any commercial or financial relationships that could be construed as a potential conflict of interest.

## Publisher’s Note

All claims expressed in this article are solely those of the authors and do not necessarily represent those of their affiliated organizations, or those of the publisher, the editors and the reviewers. Any product that may be evaluated in this article, or claim that may be made by its manufacturer, is not guaranteed or endorsed by the publisher.
