# Supplementary material for: The Redox Homeostasis of Skeletal Muscle Cells Regulates Stage Differentiation of Toxoplasma gondii
Source: Front Cell Infect Microbiol. 2021 Nov 22;11:798549. doi: 10.3389/fcimb.2021.798549 (PMC8646093; doi:10.3389/fcimb.2021.798549)
Supplement: Supplementary file 1 [file DataSheet_1.docx]

***Supplementary Material***

1. **Supplementary Figures and Tables**

**1.1 Supplementary Figures**

**
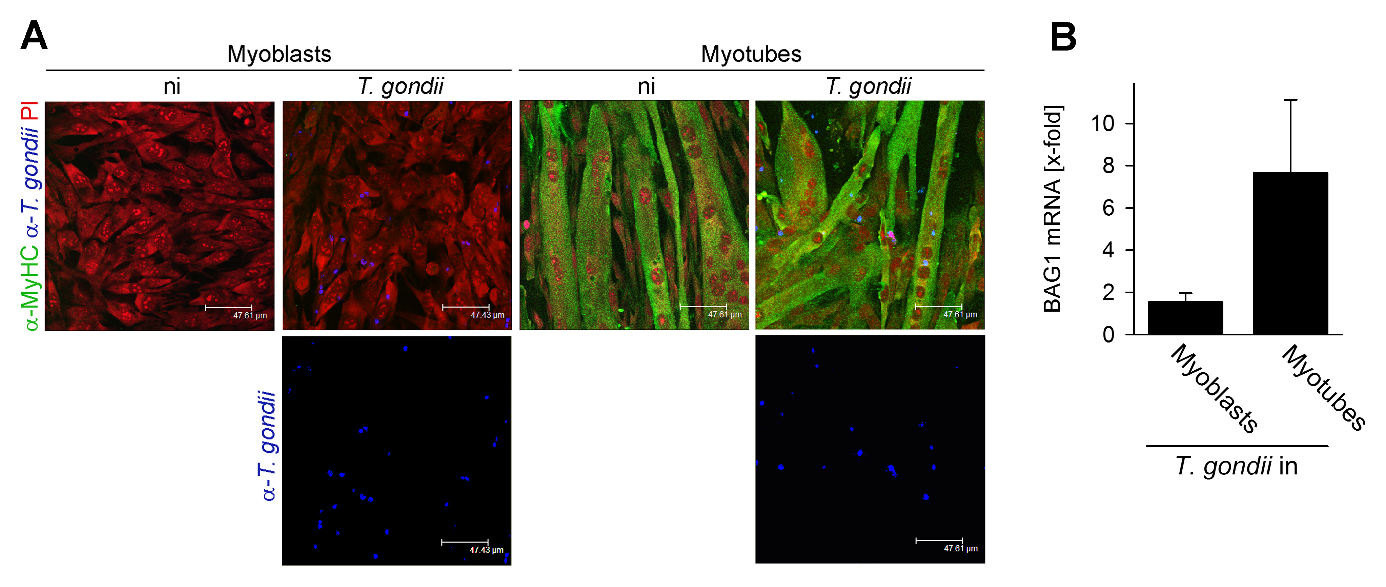
**

**Figure S1.** After differentiation *in vitro*, C2C12 SkMCs form polynucleated mature myotubes which, after infection, more readily trigger bradyzoite formation in *T. gondii* than myoblasts. C2C12 myoblasts were differentiated to multinucleated myotubes by *in vitro* cultivation in medium containing 2% horse serum. Myotubes and myoblasts were then infected with *T. gondii* at a MOI of 5:1 for 24 hours or were left non-infected. **(A)** Cells were fixed at 24 hours of infection, and were stained with anti-myosin heavy chain antibodies (green fluorescence), anti-*T. gondii* antibodies (blue fluorescence) and propidium iodide (red fluorescence). Representative images of the three labels were recorded by confocal laser scanning microscopy and were superimposed (upper panel). Labelling of *T. gondii* is also depicted separately (lower panel). **(B)** After isolation of total RNA and after reverse transcription, *T. gondii* BAG1 and *T. gondii* actin mRNAs were quantitated by real-time PCR. Bars indicate the relative expression levels of BAG1 mRNA normalized to actin mRNA (means ± S.E.M., n = 3).





**Figure S2.** Divergent expression profiles of myoblasts and myotubes and minor impact of *T. gondii* infection. C2C12 myoblasts and *in vitro*-differentiated C2C12 myotubes were infected with *T. gondii* at a MOI of 5:1 for 24 hours or were left non-infected. Total RNA from three biological replicates each were used to prepare cDNA libraries which were then sequenced using Illumina technology. After mapping the reads to the *Mus musculus* reference genome, expression profiles of individual samples were hierarchically clustered according to their overall distances.


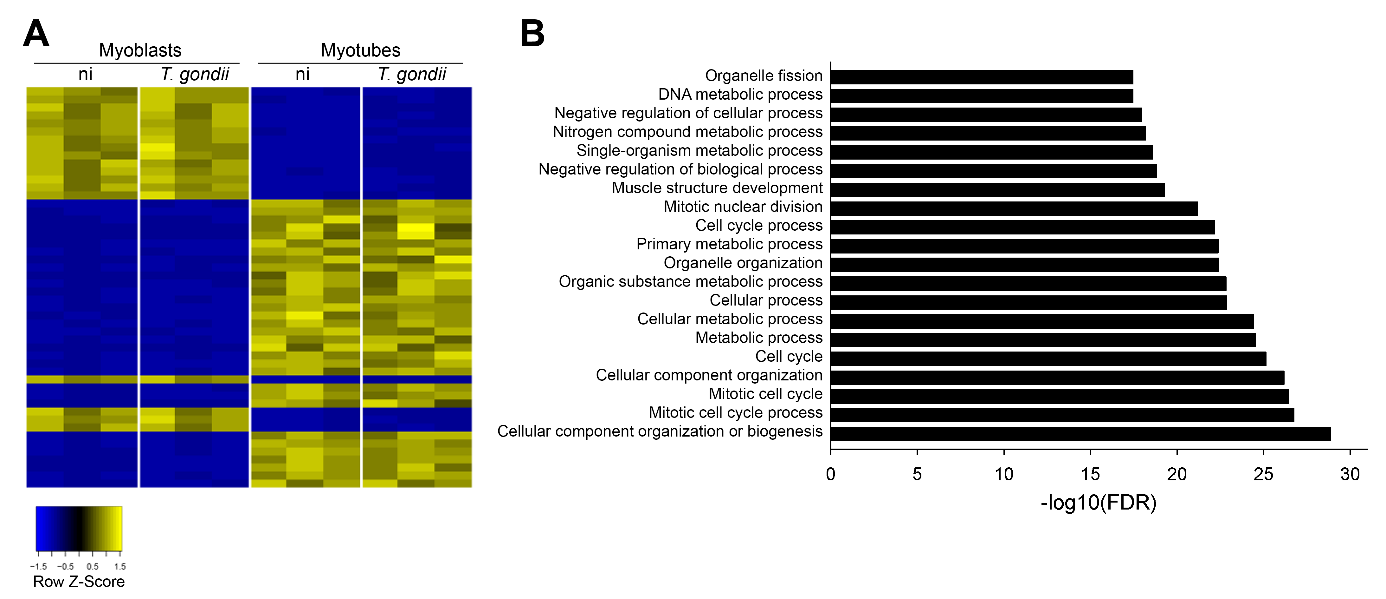


**Figure S3.** Non-infected myoblasts and myotubes differ in their transcriptome profiles. C2C12 myoblasts and *in vitro*-differentiated C2C12 myotubes were infected with *T. gondii* at a MOI of 5:1 for 24 hours or were left non-infected. Total RNA from three biological replicates each were used to prepare cDNA libraries which were then sequenced using Illumina technology. Reads were mapped to the *Mus musculus* reference genome. **(A)** The top 50 genes that were most significantly regulated between non-infected myoblasts and myotubes were hierarchically clustered. Their expression levels from three biological replicates each of non-infected (ni) and infected (*T. gondii*) myoblasts and myotubes were visualized in a heatmap. **(B)** DEGs identified between non-infected myoblasts and myotubes were functionally analyzed for enrichment of gene ontology (GO) terms. The 20 GO biological processes which were most significantly enriched are depicted.





**Figure S4.** Transcript levels and expression kinetics of multiple carbohydrate metabolic enzymes markedly differ between myoblasts and myotubes during parasite infection. C2C12 myoblasts and *in vitro*-differentiated C2C12 myotubes were infected with *T. gondii* for 4, 24 or 48 hours (Tg), or they were left non-infected (ni). After isolation of total RNA and reverse transcription, cDNAs of mouse G6PD2 **(A)**, G6PDX **(B)**, PGD **(C)** and PCX **(D)** were amplified by quantitative real-time PCR and were normalized to mouse β-actin amplicons. Bars represent the mean mRNA changes ± S.E.M. (n = 3) as compared to the mRNA levels measured in non-infected myoblasts at 4 hours of experimentation. Significant differences were identified by ANOVA (**p* < 0.05; ****p* < 0.001).





**Figure S5.** The levels of central carbon metabolism intermediates are similar in myotubes and myoblasts irrespective of *T. gondii* infection. C2C12 myoblasts and *in vitro*-differentiated myotubes were infected with *T. gondii* for 6 hours or were left non-infected and were then incubated in complete medium containing ^13^C-U-glucose for 4 hours. After cell extraction, metabolite abundances of glycolytic **(A)**, PPP **(B)** and TCA cycle metabolites **(C)** were determined by GC-MS. Values represent means ± S.E.M. of three independent experiments.

**1.2 Supplementary Tables**

**Table S1.** Expression levels of enzymes of the central carbohydrate metabolism in *T. gondii*-infected and non-infected myoblasts and myotubes.

|  |  |  | **Myoblasts** | | **Myotubes** | |  |  |
| --- | --- | --- | --- | --- | --- | --- | --- | --- |
| **Ensembl No** | **Gene ID** | **Description** | **ni^a^** | ***T. gondii*** | **ni** | ***T. gondii*** | **Ratio Mb-Tg/Mt-Tg^b^** | ***p*-value^c^** |
| **Glycolysis** | | | | | | | | |
| G00000025877 | Hk3 | hexokinase 3 | 0,17 | 0,00 | 0,00 | 0,27 | 0,000 | nd |
| G00000037012 | Hk1 | hexokinase 1 | 8795,74 | 8331,51 | 6556,25 | 6497,83 | 1,282 | 4,046E-03 |
| G00000000628 | Hk2 | hexokinase 2 | 2391,08 | 2495,12 | 3843,84 | 3874,96 | 0,644 | 1,587E-03 |
| G00000036427 | Gpi1 | glucose phosphate isomerase 1 | 9846,05 | 9601,52 | 14177,17 | 14704,36 | 0,653 | 4,267E-05 |
| G00000033065 | Pfkm^d^ | phosphofructokinase, muscle | 2274,60 | 2232,22 | 25088,74 | 24814,43 | 0,090 | 1,489E-52 |
| G00000021196 | Pfkp | phosphofructokinase, platelet | 4339,49 | 4147,98 | 3447,03 | 3568,10 | 1,163 | 3,188E-01 |
| G00000020277 | Pfkl | phosphofructokinase, liver, B-type | 9848,47 | 9333,14 | 8935,63 | 9303,23 | 1,003 | 9,845E-01 |
| G00000021456 | Fbp2 | fructose bisphosphatase 2 | 0,52 | 0,00 | 8,50 | 10,65 | 0,000 | 5,342E-07 |
| G00000069805 | Fbp1 | fructose bisphosphatase 1 | 0,17 | 0,00 | 0,00 | 0,00 | nd | nd |
| G00000030695 | Aldoa | aldolase A, fructose-bisphosphate | 35486,08 | 32972,81 | 65565,43 | 66298,06 | 0,497 | 8,358E-08 |
| G00000028307 | Aldob | aldolase B, fructose-bisphosphate | 0,00 | 0,00 | 0,45 | 1,73 | 0,000 | nd |
| G00000017390 | Aldoc | aldolase C, fructose-bisphosphate | 73,94 | 53,09 | 124,73 | 146,79 | 0,362 | 2,357E-05 |
| G00000057666 | Gapdh | glyceraldehyde-3-phosphate dehydrogenase | 690,25 | 702,93 | 682,54 | 626,26 | 1,122 | 2,615E-01 |
| G00000023456 | Tpi1 | triosephosphate isomerase 1 | 21443,78 | 20423,83 | 24513,19 | 25013,36 | 0,817 | 1,357E-01 |
| G00000062070 | Pgk1 | phosphoglycerate kinase 1 | 1929,35 | 1788,71 | 2036,55 | 2126,56 | 0,841 | 3,721E-01 |
| G00000031233 | Pgk2 | phosphoglycerate kinase 2 | 0,00 | 0,00 | 0,00 | 0,00 | nd | nd |
| G00000020475 | Pgam2 | phosphoglycerate mutase 2 | 106,22 | 83,41 | 3313,57 | 3115,43 | 0,027 | 5,118E-34 |
| G00000011752 | Pgam1 | phosphoglycerate mutase 1 | 609,20 | 595,77 | 474,63 | 501,57 | 1,188 | 2,226E-01 |
| G00000004267 | Eno2 | enolase 2, gamma neuronal | 375,85 | 360,69 | 1743,97 | 1739,46 | 0,207 | 2,342E-20 |
| G00000063524 | Eno1 | enolase 1, alpha non-neuron | 9690,33 | 8604,39 | 7764,46 | 7710,08 | 1,116 | 6,654E-01 |
| G00000048029 | Eno4 | enolase 4 | 16,92 | 20,09 | 6,01 | 5,15 | 3,903 | 4,741E-04 |
| G00000060600 | Eno3 | enolase 3, beta muscle | 19764,94 | 16469,75 | 51401,92 | 47390,09 | 0,348 | 3,337E-19 |
| G00000041237 | Pklr | pyruvate kinase liver and red blood cell | 0,00 | 0,00 | 0,00 | 0,00 | nd | nd |
| G00000032294 | Pkm | pyruvate kinase, muscle | 64899,76 | 60942,09 | 56685,11 | 56932,92 | 1,070 | 5,768E-01 |
| G00000031958 | Ldhd | lactate dehydrogenase D | 2,68 | 3,29 | 11,47 | 12,76 | 0,258 | 2,191E-03 |
| G00000063229 | Ldha | lactate dehydrogenase A | 30142,37 | 29790,87 | 30679,41 | 32008,39 | 0,931 | 6,624E-01 |
| G00000030851 | Ldhc | lactate dehydrogenase C | 0,17 | 0,26 | 0,76 | 0,00 | nd | nd |
| G00000030246 | Ldhb | lactate dehydrogenase B | 173,07 | 166,65 | 326,23 | 335,83 | 0,496 | 5,528E-05 |
| **TCA cycle** | | | | | | | | |
| G00000010914 | Pdhx | pyruvate dehydrogenase complex, component X | 921,79 | 921,90 | 1465,98 | 1439,76 | 0,640 | 4,118E-15 |
| G00000000168 | Dlat | dihydrolipoamide S-acetyltransferase | 2987,90 | 2971,88 | 3451,00 | 3410,87 | 0,871 | 1,073E-02 |
| G00000031299 | Pdha1 | pyruvate dehydrogenase E1 alpha 1 | 5281,85 | 5511,89 | 7614,27 | 7673,21 | 0,718 | 4,813E-06 |
| G00000006494 | Pdk1 | pyruvate dehydrogenase kinase, isoenzyme 1 | 1778,45 | 1800,22 | 4954,78 | 5519,25 | 0,326 | 1,173E-17 |
| G00000038967 | Pdk2 | pyruvate dehydrogenase kinase, isoenzyme 2 | 397,39 | 372,89 | 1744,64 | 1839,35 | 0,203 | 1,110E-14 |
| G00000021748 | Pdhb | pyruvate dehydrogenase (lipoamide) beta | 2060,60 | 1846,86 | 2520,18 | 2487,50 | 0,742 | 2,792E-03 |
| G00000033624 | Pdpr | pyruvate dehydrogenase phosphatase regulatory subunit | 332,37 | 497,03 | 624,13 | 713,99 | 0,696 | 6,511E-01 |
| G00000019577 | Pdk4 | pyruvate dehydrogenase kinase, isoenzyme 4 | 1448,04 | 1739,05 | 1212,92 | 1205,76 | 1,442 | 3,194E-02 |
| G00000047674 | Pdha2 | pyruvate dehydrogenase E1 alpha 2 | 0,00 | 0,00 | 0,00 | 0,00 | nd | nd |
| G00000035232 | Pdk3 | pyruvate dehydrogenase kinase, isoenzyme 3 | 1103,96 | 1051,95 | 969,17 | 940,93 | 1,118 | 1,866E-01 |
| G00000005683 | Cs | citrate synthase | 17264,04 | 17571,94 | 12121,37 | 12617,61 | 1,393 | 2,720E-08 |
| G00000028405 | Aco1 | aconitase 1 | 2654,50 | 2664,78 | 2008,52 | 1974,92 | 1,349 | 1,789E-04 |
| G00000022477 | Aco2 | aconitase 2, mitochondrial | 11676,62 | 11639,58 | 15883,56 | 15802,30 | 0,737 | 5,604E-06 |
| G00000002010 | Idh3g | isocitrate dehydrogenase 3 (NAD+), gamma | 3451,13 | 3396,40 | 4216,30 | 4203,16 | 0,808 | 2,527E-02 |
| G00000027406 | Idh3b | isocitrate dehydrogenase 3 (NAD+) beta | 3862,98 | 3738,68 | 3845,93 | 3728,25 | 1,003 | 9,793E-01 |
| G00000025950 | Idh1 | isocitrate dehydrogenase 1 (NADP+), soluble | 5893,58 | 5220,32 | 4091,94 | 3893,69 | 1,341 | 3,999E-03 |
| G00000032279 | Idh3a | isocitrate dehydrogenase 3 (NAD+) alpha | 4201,07 | 4213,88 | 3544,19 | 3572,28 | 1,180 | 1,774E-02 |
| G00000030541 | Idh2 | isocitrate dehydrogenase 2 (NADP+), mitochondrial | 7227,04 | 6722,77 | 4375,48 | 4422,57 | 1,520 | 1,399E-05 |
| G00000020456 | Ogdh | oxoglutarate (alpha-ketoglutarate) dehydrogenase | 7374,97 | 7399,76 | 7309,51 | 7317,74 | 1,011 | 9,387E-01 |
| G00000052738 | Suclg1 | succinate-CoA ligase, GDP-forming, alpha subunit | 3458,94 | 3147,43 | 3950,17 | 3799,33 | 0,828 | 1,908E-01 |
| G00000009863 | Sdhb | succinate dehydrogenase complex, subunit B | 4258,72 | 3923,32 | 3445,92 | 3322,18 | 1,181 | 3,638E-01 |
| G00000026154 | Sdhaf4 | succinate dehydrogenase complex assembly factor 4 | 426,08 | 375,22 | 493,63 | 495,55 | 0,757 | 1,130E-01 |
| G00000058076 | Sdhc | succinate dehydrogenase complex, subunit C | 4898,84 | 4382,56 | 5051,42 | 4872,76 | 0,899 | 4,580E-01 |
| G00000024668 | Sdhaf2 | succinate dehydrogenase complex assembly factor 2 | 1682,86 | 1633,55 | 1672,33 | 1674,30 | 0,976 | 8,468E-01 |
| G00000021577 | Sdha | succinate dehydrogenase complex, subunit A | 7870,85 | 7644,87 | 8532,70 | 8158,17 | 0,937 | 3,572E-01 |
| G00000000171 | Sdhd | succinate dehydrogenase complex, subunit D | 4624,57 | 4411,99 | 4040,00 | 4048,15 | 1,090 | 5,218E-01 |
| G00000074211 | Sdhaf1 | succinate dehydrogenase complex assembly factor 1 | 61,52 | 80,74 | 91,55 | 102,02 | 0,791 | 2,204E-01 |
| G00000042505 | Sdhaf3 | succinate dehydrogenase complex assembly factor 3 | 144,85 | 137,39 | 246,77 | 250,44 | 0,549 | 2,746E-07 |
| G00000026526 | Fh1 | fumarate hydratase 1 | 4211,05 | 4092,49 | 3532,83 | 3600,27 | 1,137 | 1,976E-01 |
| G00000020321 | Mdh1 | malate dehydrogenase 1, NAD (soluble) | 6151,97 | 5529,03 | 8386,34 | 8149,61 | 0,678 | 8,721E-04 |
| G00000019179 | Mdh2 | malate dehydrogenase 2, NAD (mitochondrial) | 15547,65 | 14652,56 | 12400,33 | 12079,20 | 1,213 | 2,145E-01 |
| G00000025963 | Mdh1b | malate dehydrogenase 1B, NAD (soluble) | 3,17 | 1,84 | 4,71 | 2,72 | 0,677 | 8,249E-01 |
| **TCA cycle anaplerosis** | | | | | | | | |
| G00000024892 | Pcx | pyruvate carboxylase | 497,34 | 464,49 | 1759,70 | 1622,82 | 0,286 | 7,055E-14 |
| G00000040618 | Pck2 | phosphoenolpyruvate carboxykinase 2 (mitochondrial) | 3550,36 | 3483,44 | 1590,14 | 1703,32 | 2,045 | 3,471E-11 |
| G00000027513 | Pck1 | phosphoenolpyruvate carboxykinase 1, cytosolic | 0,00 | 0,36 | 0,76 | 0,00 | nd | nd |
| G00000024556 | Me2 | malic enzyme 2, NAD(+)-dependent, mitochondrial | 3271,72 | 3286,57 | 1512,21 | 1525,69 | 2,154 | 9,798E-16 |
| G00000032418 | Me1 | malic enzyme 1, NADP(+)-dependent, cytosolic | 2281,84 | 2158,98 | 1484,94 | 1429,57 | 1,510 | 1,271E-05 |
| G00000030621 | Me3 | malic enzyme 3, NADP(+)-dependent, mitochondrial | 74,06 | 54,95 | 438,00 | 416,67 | 0,132 | 1,585E-33 |
| **Pentose phosphate pathway** | | | | | | | | |
| G00000089992 | G6pd2 | glucose-6-phosphate dehydrogenase 2 | 0,69 | 5,03 | 0,00 | 0,88 | 5,706 | 4,199E-02 |
| G00000031400 | G6pdx | glucose-6-phosphate dehydrogenase X-linked | 4002,39 | 4319,00 | 1151,64 | 1161,26 | 3,719 | 8,529E-61 |
| G00000028980 | H6pd | hexose-6-phosphate dehydrogenase | 1328,65 | 1406,77 | 1664,77 | 1712,06 | 0,822 | 1,241E-01 |
| G00000028961 | Pgd | phosphogluconate dehydrogenase | 10217,70 | 9879,02 | 4508,71 | 4259,35 | 2,319 | 2,470E-13 |
| G00000031807 | Pgls | 6-phosphogluconolactonase | 2714,66 | 2355,19 | 2285,01 | 2288,07 | 1,029 | 9,178E-01 |
| G00000053604 | Rpia | ribose 5-phosphate isomerase A | 628,25 | 619,27 | 496,18 | 482,39 | 1,284 | 1,680E-02 |
| G00000026005 | Rpe | ribulose-5-phosphate-3-epimerase | 1000,11 | 1105,00 | 754,29 | 771,33 | 1,433 | 5,067E-06 |
| G00000021957 | Tkt | transketolase | 2828,63 | 2828,78 | 1049,58 | 1124,96 | 2,515 | 3,299E-08 |
| G00000025503 | Taldo1 | transaldolase 1 | 4717,46 | 4097,25 | 3507,40 | 3399,36 | 1,205 | 3,254E-01 |
| **Glycogen metabolism** | | | | | | | | |
| G00000041731 | Pgm5 | phosphoglucomutase 5 | 191,70 | 201,75 | 9119,77 | 9405,04 | 0,021 | 1,784E-12 |
| G00000056131 | Pgm3 | phosphoglucomutase 3 | 544,40 | 562,50 | 590,69 | 547,65 | 1,027 | 8,090E-01 |
| G00000030729 | Pgm2l1 | phosphoglucomutase 2-like 1 | 177,66 | 208,89 | 72,12 | 88,20 | 2,368 | 1,143E-06 |
| G00000029171 | Pgm1 | phosphoglucomutase 1 | 1676,53 | 1628,38 | 824,64 | 787,67 | 2,067 | 5,632E-19 |
| G00000025791 | Pgm2 | phosphoglucomutase 2 | 3962,40 | 3599,71 | 5382,58 | 5333,62 | 0,675 | 3,927E-03 |
| G00000001891 | Ugp2 | UDP-glucose pyrophosphorylase 2 | 3400,56 | 3088,91 | 4236,06 | 4268,95 | 0,724 | 4,514E-05 |
| G00000003865 | Gys1 | glycogen synthase 1, muscle | 1951,76 | 1837,02 | 8084,67 | 8069,19 | 0,228 | 1,489E-52 |
| G00000030244 | Gys2 | glycogen synthase 2 | 0,00 | 0,62 | 0,00 | 0,46 | 1,356 | nd |
| G00000019528 | Gyg | glycogenin | 1653,29 | 1518,17 | 4430,68 | 4441,82 | 0,342 | 1,673E-35 |
| G00000022707 | Gbe1 | glucan (1,4-alpha-), branching enzyme 1 | 687,60 | 723,33 | 764,15 | 809,71 | 0,893 | 2,819E-01 |
| G00000032648 | Pygm | muscle glycogen phosphorylase | 229,02 | 235,54 | 8609,46 | 8544,74 | 0,028 | 3,956E-14 |
| G00000021069 | Pygl | liver glycogen phosphorylase | 1,83 | 2,50 | 6,76 | 8,83 | 0,283 | 3,176E-02 |
| G00000033059 | Pygb | brain glycogen phosphorylase | 2441,94 | 2449,73 | 1702,18 | 1667,96 | 1,469 | 2,988E-06 |
| G00000032469 | Dbr1 | debranching enzyme homolog 1 (S. cerevisiae) | 715,89 | 724,94 | 399,21 | 418,07 | 1,734 | 6,987E-08 |
| G00000033400 | Agl | amylo-1,6-glucosidase, 4-alpha-glucanotransferase | 1796,73 | 1872,06 | 4833,24 | 4946,82 | 0,378 | 3,020E-09 |
| ^a^Data are mean normalized reads (n = 3) as determined by RNA sequencing. | | | | | | | | |
| ^b^Data are ratios of mean expression levels between infected myoblasts (Mb-Tg) versus infected myotubes (Mt-Tg). | | | | | | | | |
| ^c^FDR-corrected *p*-value. | | | | | | | | |
| ^d^Color code: In myoblasts up-regulated; in myotubes up-regulated. | | |  |  |  |  |  |  |

**Table S2.** Expression levels of Nrf2 target genes in *T. gondii*-infected and non-infected myoblasts and myotubes.

|  |  | **Myoblasts** | | **Myotubes** | |  |  |
| --- | --- | --- | --- | --- | --- | --- | --- |
| **Gene ID** | **Description** | **ni^a^** | ***T. gondii*** | **ni** | ***T. gondii*** | **Ratio Mb-Tg/Mt-Tg^b^** | ***p*-value^c^** |
| **Nrf2-basal+inducible** | | | | | | | |
| Nqo1 | NAD(P)H dehydrogenase, quinone 1^d^ | 1861,16 | 1626,52 | 188,38 | 190,78 | 8,526 | 2,900E-28 |
| Gsta4 | glutathione S-transferase, alpha 4 | 4345,71 | 4205,70 | 567,50 | 535,62 | 7,852 | 7,089E-41 |
| Foxa2 | forkhead box A2 | 20,11 | 13,34 | 5,08 | 2,62 | 5,100 | 4,360E-03 |
| Srxn1 | sulfiredoxin 1 homolog (S. cerevisiae) | 3579,11 | 3397,92 | 938,29 | 882,97 | 3,848 | 3,218E-45 |
| G6pdx | glucose-6-phosphate dehydrogenase X-linked | 4002,39 | 4319,00 | 1151,64 | 1161,26 | 3,719 | 8,529E-61 |
| Cxcl5 | chemokine (C-X-C motif) ligand 5 | 260,58 | 219,65 | 67,77 | 65,02 | 3,378 | 2,673E-09 |
| Txnrd1 | thioredoxin reductase 1 | 13759,79 | 14926,89 | 4683,24 | 4793,77 | 3,114 | 1,812E-31 |
| Slc39a10 | solute carrier family 39 (zinc transporter), member 10 | 1037,18 | 1355,10 | 437,74 | 493,11 | 2,748 | 6,966E-08 |
| Prkar2b | protein kinase, cAMP dependent regulatory, type II beta | 3349,84 | 3318,98 | 1105,80 | 1214,04 | 2,734 | 9,472E-25 |
| Dlx2 | distal-less homeobox 2 | 168,93 | 186,11 | 64,97 | 70,67 | 2,633 | 1,238E-08 |
| Rps6 | ribosomal protein S6 | 122,38 | 121,19 | 47,84 | 47,02 | 2,577 | 2,351E-05 |
| Tkt | transketolase | 2828,63 | 2828,78 | 1049,58 | 1124,96 | 2,515 | 3,299E-08 |
| Mmd | monocyte to macrophage differentiation-associated | 6893,95 | 6823,96 | 3007,48 | 3044,76 | 2,241 | 1,575E-29 |
| Ccdc109b | coiled-coil domain containing 109B | 1419,91 | 1195,54 | 537,31 | 546,98 | 2,186 | 9,905E-07 |
| Rgs10 | regulator of G-protein signalling 10 | 269,91 | 249,53 | 115,47 | 114,91 | 2,172 | 5,103E-04 |
| Txn1 | thioredoxin 1 | 18126,33 | 16260,86 | 7925,76 | 7886,03 | 2,062 | 4,815E-05 |
| Magohb | mago-nashi homolog B (Drosophila) | 612,37 | 550,11 | 273,29 | 270,31 | 2,035 | 9,984E-06 |
| Pspc1 | paraspeckle protein 1 | 1316,24 | 1318,38 | 714,55 | 664,01 | 1,985 | 6,869E-18 |
| Lpl | lipoprotein lipase | 2422,47 | 2289,65 | 1115,78 | 1166,77 | 1,962 | 1,036E-02 |
| Anxa1 | annexin A1 | 34626,15 | 31909,42 | 16775,60 | 16470,00 | 1,937 | 9,457E-08 |
| Adam23 | a disintegrin and metallopeptidase domain 23 | 136,21 | 141,99 | 74,38 | 74,59 | 1,904 | 2,113E-04 |
| Gclc | glutamate-cysteine ligase, catalytic subunit | 1655,76 | 1875,95 | 1021,54 | 987,76 | 1,899 | 2,171E-05 |
| Ptgr1 | prostaglandin reductase 1 | 3791,74 | 3261,89 | 1948,19 | 1802,99 | 1,809 | 6,372E-05 |
| Wasf1 | WAS protein family, member 1 | 738,99 | 736,10 | 456,35 | 409,38 | 1,798 | 8,415E-15 |
| Rpl10a | ribosomal protein L10A | 966,67 | 1069,82 | 592,24 | 597,59 | 1,790 | 7,491E-08 |
| Kras | v-Ki-ras2 Kirsten rat sarcoma viral oncogene homolog | 4834,55 | 4900,08 | 2649,01 | 2792,63 | 1,755 | 2,168E-18 |
| Arhgap18 | Rho GTPase activating protein 18 | 934,92 | 956,28 | 530,29 | 547,26 | 1,747 | 3,494E-09 |
| Cbr3 | carbonyl reductase 3 | 592,26 | 589,30 | 350,37 | 338,03 | 1,743 | 1,205E-03 |
| Gstm1 | glutathione S-transferase, mu 1 | 2613,61 | 2472,73 | 1463,70 | 1483,63 | 1,667 | 1,449E-04 |
| Acap2 | ArfGAP with coiled-coil, ankyrin repeat and PH domains 2 | 2313,48 | 2528,47 | 1540,01 | 1585,30 | 1,595 | 1,170E-02 |
| Pla2g4a | phospholipase A2, group IVA (cytosolic, calcium-dependent) | 936,90 | 887,50 | 577,54 | 558,08 | 1,590 | 8,259E-05 |
| Fzd6 | frizzled homolog 6 (Drosophila) | 1128,61 | 1142,80 | 773,67 | 730,02 | 1,565 | 2,070E-04 |
| Ephx1 | epoxide hydrolase 1, microsomal | 3492,62 | 3699,70 | 2298,15 | 2367,29 | 1,563 | 8,516E-05 |
| Prdx1 | peroxiredoxin 1 | 10439,35 | 9527,53 | 6348,79 | 6109,92 | 1,559 | 4,501E-03 |
| Phlda1 | pleckstrin homology-like domain, family A, member 1 | 5777,12 | 5236,98 | 3455,51 | 3466,73 | 1,511 | 4,690E-03 |
| Prr13 | proline rich 13 | 4459,75 | 3876,22 | 2766,54 | 2575,47 | 1,505 | 3,643E-03 |
| Cltc | clathrin, heavy polypeptide (Hc) | 12653,75 | 13668,88 | 9488,06 | 9120,26 | 1,499 | 2,870E-02 |
| Prkcd | protein kinase C, delta | 1453,99 | 1480,51 | 978,91 | 1004,35 | 1,474 | 6,581E-09 |
| Cpt1a | carnitine palmitoyltransferase 1a, liver | 2014,91 | 2082,89 | 1457,75 | 1422,63 | 1,464 | 1,224E-02 |
| Sema6d | sema domain, transmembrane domain (TM), and cytoplasmic domain, (semaphorin) 6D | 77,07 | 100,13 | 62,85 | 68,61 | 1,459 | 4,185E-02 |
| Ado | 2-aminoethanethiol (cysteamine) dioxygenase | 3409,98 | 3334,87 | 2183,45 | 2319,30 | 1,438 | 6,406E-07 |
| Mapk8 | mitogen-activated protein kinase 8 | 1135,38 | 1167,10 | 1619,75 | 1664,40 | 0,701 | 1,386E-08 |
| Bbs9 | Bardet-Biedl syndrome 9 (human) | 285,03 | 266,77 | 369,63 | 381,78 | 0,699 | 1,228E-03 |
| Pttg1ip | pituitary tumor-transforming 1 interacting protein | 7751,06 | 7518,11 | 11417,31 | 10855,46 | 0,693 | 1,504E-06 |
| Ccdc90b | coiled-coil domain containing 90B | 522,55 | 486,20 | 713,36 | 714,45 | 0,681 | 7,296E-05 |
| Tmem159 | transmembrane protein 159 | 435,83 | 394,46 | 625,82 | 587,20 | 0,672 | 1,534E-03 |
| Tbcel | tubulin folding cofactor E-like | 794,06 | 826,93 | 1222,82 | 1234,89 | 0,670 | 1,700E-03 |
| Tmem64 | transmembrane protein 64 | 7316,11 | 6966,13 | 10502,74 | 10403,01 | 0,670 | 8,162E-03 |
| Rnd3 | Rho family GTPase 3 | 1337,36 | 1253,50 | 1927,48 | 1878,92 | 0,667 | 2,099E-11 |
| Rxra | retinoid X receptor alpha | 1529,29 | 1611,16 | 2479,89 | 2479,08 | 0,650 | 7,356E-07 |
| Appl2 | adaptor protein, phosphotyrosine interaction, PH domain and leucine zipper containing 2 | 620,18 | 620,62 | 1035,82 | 966,40 | 0,642 | 7,340E-04 |
| Insig2 | insulin induced gene 2 | 2036,52 | 1843,70 | 2907,17 | 2878,54 | 0,640 | 2,365E-06 |
| Megf9 | multiple EGF-like-domains 9 | 544,90 | 615,30 | 906,04 | 972,06 | 0,633 | 2,558E-02 |
| Vps8 | vacuolar protein sorting 8 homolog (S. cerevisiae) | 968,39 | 990,11 | 1613,90 | 1572,46 | 0,630 | 2,187E-08 |
| Tbc1d23 | TBC1 domain family, member 23 | 966,05 | 954,82 | 1562,37 | 1533,19 | 0,623 | 3,793E-05 |
| Heca | headcase homolog (Drosophila) | 449,13 | 503,61 | 773,39 | 815,25 | 0,618 | 3,171E-02 |
| Fkbpl | FK506 binding protein-like | 270,87 | 217,13 | 384,87 | 351,81 | 0,617 | 2,645E-03 |
| Scamp1 | secretory carrier membrane protein 1 | 1376,16 | 1290,09 | 2154,44 | 2139,75 | 0,603 | 8,012E-08 |
| Snx22 | sorting nexin 22 | 29,31 | 21,04 | 57,82 | 39,77 | 0,529 | 2,661E-03 |
| Coro7 | coronin 7 | 356,78 | 353,04 | 699,21 | 677,48 | 0,521 | 1,988E-06 |
| Ypel5 | yippee-like 5 (Drosophila) | 1442,80 | 1183,00 | 2378,74 | 2271,60 | 0,521 | 1,028E-05 |
| Cpne8 | copine VIII | 568,92 | 598,95 | 1200,32 | 1163,61 | 0,515 | 1,811E-10 |
| Chpt1 | choline phosphotransferase 1 | 1193,13 | 1167,53 | 2272,79 | 2269,02 | 0,515 | 2,254E-15 |
| Ctso | cathepsin O | 348,90 | 333,57 | 685,74 | 656,94 | 0,508 | 4,082E-08 |
| Ahr | aryl-hydrocarbon receptor | 399,25 | 569,05 | 1075,91 | 1129,81 | 0,504 | 2,916E-05 |
| 2010111I01Rik | RIKEN cDNA 2010111I01 gene | 1590,08 | 1388,73 | 3347,09 | 3065,31 | 0,453 | 2,166E-09 |
| Tmtc2 | transmembrane and tetratricopeptide repeat containing 2 | 67,16 | 65,14 | 147,05 | 148,02 | 0,440 | 7,669E-03 |
| Hbp1 | high mobility group box transcription factor 1 | 1735,74 | 1760,37 | 4215,86 | 4093,14 | 0,430 | 1,064E-20 |
| Mlycd | malonyl-CoA decarboxylase | 355,80 | 326,97 | 753,83 | 772,27 | 0,423 | 3,522E-09 |
| Tspan3 | tetraspanin 3 | 3279,54 | 3071,86 | 7963,40 | 7760,47 | 0,396 | 6,194E-30 |
| Cdh11 | cadherin 11 | 456,53 | 475,72 | 1268,60 | 1226,26 | 0,388 | 1,923E-05 |
| Map3k9 | mitogen-activated protein kinase 9 | 43,08 | 45,67 | 126,66 | 132,16 | 0,346 | 5,105E-05 |
| Kcnab1 | potassium voltage-gated channel, shaker-related subfamily, beta member 1 | 17,44 | 14,90 | 35,06 | 43,23 | 0,345 | 1,592E-02 |
| Npnt | nephronectin | 8225,66 | 7806,93 | 22547,17 | 23245,56 | 0,336 | 3,058E-21 |
| Tgfb2 | transforming growth factor, beta 2 | 696,47 | 743,68 | 2426,01 | 2334,61 | 0,319 | 1,229E-19 |
| Kif26b | kinesin family member 26B | 78,03 | 80,42 | 310,96 | 266,05 | 0,302 | 8,948E-07 |
| Bmper | BMP-binding endothelial regulator | 329,52 | 305,62 | 1209,24 | 1106,33 | 0,276 | 1,739E-15 |
| Cd302 | CD302 antigen | 116,14 | 104,34 | 402,29 | 378,00 | 0,276 | 1,894E-13 |
| Ddc | dopa decarboxylase | 36,59 | 37,77 | 140,56 | 155,31 | 0,243 | 2,833E-10 |
| Ppp1r12b | protein phosphatase 1, regulatory (inhibitor) subunit 12B | 255,65 | 301,56 | 1323,42 | 1357,62 | 0,222 | 1,393E-10 |
| Gli2 | GLI-Kruppel family member GLI2 | 4,27 | 2,66 | 12,91 | 14,01 | 0,190 | 8,804E-04 |
| Dcn | decorin | 632,76 | 631,65 | 3504,71 | 3577,46 | 0,177 | 1,190E-04 |
| Synpo2 | synaptopodin 2 | 1464,39 | 1509,86 | 12776,61 | 12422,23 | 0,122 | 5,377E-31 |
| Daam2 | dishevelled associated activator of morphogenesis 2 | 440,68 | 420,82 | 3505,57 | 3503,90 | 0,120 | 1,953E-13 |
| Bmp8a | bone morphogenetic protein 8a | 4,60 | 5,03 | 51,74 | 42,80 | 0,118 | 3,427E-09 |
| Igf1 | insulin-like growth factor 1 | 159,41 | 139,04 | 1344,34 | 1203,99 | 0,115 | 1,548E-132 |
| Mamdc2 | MAM domain containing 2 | 650,65 | 662,86 | 5604,30 | 5850,97 | 0,113 | 8,192E-33 |
| Slc44a3 | solute carrier family 44, member 3 | 3,20 | 2,04 | 18,45 | 21,71 | 0,094 | 6,513E-08 |
| Grip2 | glutamate receptor interacting protein 2 | 14,47 | 12,24 | 194,96 | 192,80 | 0,063 | 6,361E-12 |
| Txlnb | taxilin beta | 73,97 | 77,62 | 2683,59 | 2913,40 | 0,027 | 4,113E-33 |
|  |  |  |  |  |  |  |  |
| **Nrf2-inducible** | | | | | | | |
| Prl2c2 | prolactin family 2, subfamily c, member 2 | 345,95 | 284,73 | 2,81 | 1,88 | 151,055 | 1,142E-28 |
| Oasl1 | 2'-5' oligoadenylate synthetase-like 1 | 15,70 | 12,72 | 0,76 | 0,31 | 41,656 | 6,454E-05 |
| Prl2c5 | prolactin family 2, subfamily c, member 5 | 95,20 | 85,53 | 1,91 | 3,77 | 22,688 | 7,929E-14 |
| Lrp2 | low density lipoprotein receptor-related protein 2 | 20,23 | 20,18 | 2,35 | 1,07 | 18,848 | 8,734E-03 |
| Lrp8 | low density lipoprotein receptor-related protein 8, apolipoprotein e receptor | 1235,33 | 1609,12 | 164,95 | 193,35 | 8,322 | 1,449E-32 |
| Asgr1 | asialoglycoprotein receptor 1 | 48,01 | 41,44 | 4,62 | 6,29 | 6,589 | 5,805E-10 |
| Plaur | plasminogen activator, urokinase receptor | 3242,73 | 2771,14 | 433,31 | 456,40 | 6,072 | 3,057E-25 |
| Pctp | phosphatidylcholine transfer protein | 66,28 | 75,46 | 10,92 | 14,05 | 5,370 | 8,295E-09 |
| Gsta3 | glutathione S-transferase, alpha 3 | 4,84 | 6,28 | 1,53 | 1,19 | 5,266 | 3,442E-02 |
| Pawr | PRKC, apoptosis, WT1, regulator | 29,73 | 28,67 | 5,02 | 5,94 | 4,826 | 2,859E-04 |
| Slc7a11 | solute carrier family 7 (cationic amino acid transporter, y+ system), member 11 | 124,45 | 142,48 | 30,54 | 30,25 | 4,711 | 1,326E-14 |
| Abcc4 | ATP-binding cassette, sub-family C (CFTR/MRP), member 4 | 717,87 | 803,42 | 172,22 | 175,17 | 4,587 | 1,209E-25 |
| Ckap2l | cytoskeleton associated protein 2-like | 3383,33 | 3536,38 | 763,15 | 838,63 | 4,217 | 5,364E-47 |
| Dusp4 | dual specificity phosphatase 4 | 3237,77 | 3466,72 | 910,15 | 903,82 | 3,836 | 6,154E-11 |
| Ftl1 | ferritin light chain 1 | 7465,77 | 6948,69 | 1966,45 | 1836,56 | 3,784 | 2,562E-30 |
| Cachd1 | cache domain containing 1 | 602,59 | 743,91 | 190,36 | 198,11 | 3,755 | 6,823E-20 |
| Gpr85 | G protein-coupled receptor 85 | 26,96 | 19,03 | 5,76 | 5,52 | 3,448 | 4,190E-02 |
| Dctd | dCMP deaminase | 563,93 | 762,62 | 204,01 | 222,46 | 3,428 | 4,729E-26 |
| Ppap2c | phosphatidic acid phosphatase type 2C | 5555,57 | 4934,95 | 1506,84 | 1469,36 | 3,359 | 1,874E-14 |
| Man1a | mannosidase 1, alpha | 649,61 | 733,43 | 210,58 | 230,98 | 3,175 | 1,667E-15 |
| Mfsd7b | major facilitator superfamily domain containing 7B | 589,60 | 614,95 | 217,21 | 198,05 | 3,105 | 3,836E-18 |
| Cdkn3 | cyclin-dependent kinase inhibitor 3 | 359,04 | 290,91 | 99,03 | 100,44 | 2,896 | 2,247E-06 |
| Efna5 | ephrin A5 | 56,96 | 71,83 | 24,66 | 25,33 | 2,836 | 1,037E-02 |
| Gja1 | gap junction protein, alpha 1 | 6208,65 | 7025,84 | 2252,48 | 2485,54 | 2,827 | 1,014E-30 |
| Kitl | kit ligand | 1588,89 | 1678,15 | 579,26 | 607,38 | 2,763 | 4,556E-05 |
| Etaa1 | Ewing tumor-associated antigen 1 | 654,77 | 685,84 | 237,03 | 253,38 | 2,707 | 8,259E-29 |
| Gclm | glutamate-cysteine ligase, modifier subunit | 2338,69 | 2300,15 | 885,28 | 852,57 | 2,698 | 9,739E-56 |
| Avpr1a | arginine vasopressin receptor 1A | 71,49 | 46,50 | 12,81 | 17,38 | 2,676 | 2,127E-02 |
| Mtap | methylthioadenosine phosphorylase | 6707,07 | 7182,20 | 2482,67 | 2694,55 | 2,665 | 6,022E-40 |
| D1Ertd622e | DNA segment, Chr 1, ERATO Doi 622, expressed | 861,75 | 910,77 | 357,23 | 353,29 | 2,578 | 9,046E-19 |
| Efnb2 | ephrin B2 | 2991,75 | 2931,49 | 1267,45 | 1231,67 | 2,380 | 1,585E-18 |
| Ncoa7 | nuclear receptor coactivator 7 | 1361,60 | 1469,18 | 601,28 | 627,53 | 2,341 | 4,539E-10 |
| Pgd | phosphogluconate dehydrogenase | 10217,70 | 9879,02 | 4508,71 | 4259,35 | 2,319 | 2,470E-13 |
| Epb4.1 | erythrocyte protein band 4.1 | 828,09 | 942,98 | 351,81 | 418,62 | 2,253 | 4,455E-07 |
| Adh7 | alcohol dehydrogenase 7 (class IV), mu or sigma polypeptide | 1301,36 | 1398,82 | 596,91 | 629,01 | 2,224 | 6,856E-29 |
| Rhou | ras homolog gene family, member U | 2370,12 | 2190,83 | 1000,15 | 1005,93 | 2,178 | 1,739E-07 |
| Ppif | peptidylprolyl isomerase F (cyclophilin F) | 785,39 | 754,33 | 311,52 | 354,81 | 2,126 | 8,054E-09 |
| Igf2bp2 | insulin-like growth factor 2 mRNA binding protein 2 | 1960,49 | 2077,65 | 973,24 | 980,49 | 2,119 | 1,226E-15 |
| Slpi | secretory leukocyte peptidase inhibitor | 54,70 | 66,83 | 37,17 | 33,47 | 1,997 | 4,692E-03 |
| Ptgir | prostaglandin I receptor (IP) | 245,85 | 278,15 | 127,33 | 139,45 | 1,995 | 8,024E-04 |
| St3gal1 | ST3 beta-galactoside alpha-2,3-sialyltransferase 1 | 5068,09 | 4992,17 | 2499,19 | 2511,00 | 1,988 | 5,012E-10 |
| Coq7 | demethyl-Q 7 | 776,46 | 713,90 | 369,82 | 361,78 | 1,973 | 9,849E-04 |
| Gdnf | glial cell line derived neurotrophic factor | 479,19 | 503,05 | 245,69 | 255,76 | 1,967 | 1,345E-09 |
| Zc3hav1 | zinc finger CCCH type, antiviral 1 | 2783,96 | 3050,34 | 1508,01 | 1559,04 | 1,957 | 1,098E-08 |
| Ywhaz | tyrosine 3-monooxygenase/tryptophan 5-monooxygenase activation protein, zeta polypeptide | 16849,12 | 14132,56 | 7535,43 | 7245,09 | 1,951 | 3,576E-04 |
| Slc30a4 | solute carrier family 30 (zinc transporter), member 4 | 7119,83 | 7525,51 | 3871,44 | 4035,79 | 1,865 | 4,008E-09 |
| Fscn1 | fascin homolog 1, actin bundling protein (Strongylocentrotus purpuratus) | 22196,85 | 21153,35 | 11855,63 | 11443,57 | 1,848 | 2,284E-08 |
| Fdft1 | farnesyl diphosphate farnesyl transferase 1 | 2552,76 | 2400,88 | 1403,03 | 1315,31 | 1,825 | 1,029E-08 |
| Chn2 | chimerin 2 | 812,12 | 778,87 | 427,17 | 426,95 | 1,824 | 2,543E-07 |
| Esd | esterase D/formylglutathione hydrolase | 10959,82 | 10072,48 | 5802,12 | 5546,34 | 1,816 | 6,305E-05 |
| Fancl | Fanconi anemia, complementation group L | 422,46 | 398,20 | 218,45 | 219,37 | 1,815 | 5,235E-11 |
| Cep70 | centrosomal protein 70 | 340,05 | 336,04 | 199,74 | 185,47 | 1,812 | 9,795E-11 |
| Coil | coilin | 446,75 | 492,86 | 271,66 | 273,05 | 1,805 | 1,165E-08 |
| Lamc2 | laminin, gamma 2 | 754,17 | 717,85 | 443,53 | 404,88 | 1,773 | 2,508E-05 |
| Pou2f1 | POU domain, class 2, transcription factor 1 | 555,28 | 726,87 | 370,57 | 419,82 | 1,731 | 3,191E-02 |
| Frmd8 | FERM domain containing 8 | 3050,61 | 2816,37 | 1736,57 | 1627,63 | 1,730 | 1,241E-15 |
| Rpl5 | ribosomal protein L5 | 98,00 | 112,26 | 59,59 | 66,93 | 1,677 | 1,987E-03 |
| Fbxo33 | F-box protein 33 | 412,02 | 463,11 | 308,88 | 280,36 | 1,652 | 2,594E-06 |
| Fetub | fetuin beta | 165,55 | 167,68 | 83,35 | 101,70 | 1,649 | 1,795E-03 |
| Hsd17b7 | hydroxysteroid (17-beta) dehydrogenase 7 | 1101,79 | 1184,08 | 729,67 | 720,50 | 1,643 | 7,604E-04 |
| Cstb | cystatin B | 3574,37 | 3686,98 | 2270,70 | 2244,46 | 1,643 | 8,259E-04 |
| Psmg1 | proteasome (prosome, macropain) assembly chaperone 1 | 1119,18 | 1092,74 | 754,75 | 678,54 | 1,610 | 1,976E-04 |
| Add3 | adducin 3 (gamma) | 1217,28 | 1312,11 | 769,14 | 818,44 | 1,603 | 5,042E-04 |
| Mtmr12 | myotubularin related protein 12 | 1024,79 | 1132,67 | 672,84 | 708,97 | 1,598 | 5,501E-04 |
| Aacs | acetoacetyl-CoA synthetase | 2599,80 | 2529,10 | 1647,92 | 1587,45 | 1,593 | 1,298E-10 |
| Map3k1 | mitogen-activated protein kinase 1 | 159,80 | 189,25 | 141,27 | 120,39 | 1,572 | 1,558E-02 |
| Adat2 | adenosine deaminase, tRNA-specific 2 | 249,92 | 253,19 | 146,95 | 161,10 | 1,572 | 1,722E-04 |
| Sp3 | trans-acting transcription factor 3 | 2853,43 | 3144,79 | 1952,63 | 2012,57 | 1,563 | 3,375E-05 |
| Dhx37 | DEAH (Asp-Glu-Ala-His) box polypeptide 37 | 1331,06 | 1501,53 | 921,19 | 962,89 | 1,559 | 1,311E-04 |
| D19Bwg1357e | DNA segment, Chr 19, Brigham & Women's Genetics 1357 expressed | 3573,38 | 3870,28 | 2406,98 | 2489,86 | 1,554 | 8,238E-06 |
| Mreg | melanoregulin | 184,77 | 192,02 | 113,34 | 124,12 | 1,547 | 4,929E-03 |
| Ipo9 | importin 9 | 6603,09 | 7088,56 | 4624,59 | 4664,07 | 1,520 | 2,890E-04 |
| Tbc1d14 | TBC1 domain family, member 14 | 1401,76 | 1521,88 | 1017,82 | 1009,27 | 1,508 | 8,725E-05 |
| Rasl11b | RAS-like, family 11, member B | 182,79 | 175,61 | 136,10 | 116,60 | 1,506 | 4,255E-02 |
| Ets1 | E26 avian leukemia oncogene 1, 5' domain | 1352,08 | 1501,67 | 1014,81 | 1002,62 | 1,498 | 5,721E-04 |
| Btbd3 | BTB (POZ) domain containing 3 | 512,75 | 570,58 | 364,17 | 383,35 | 1,488 | 1,520E-03 |
| Smc5 | structural maintenance of chromosomes 5 | 2697,73 | 2905,53 | 1935,90 | 1977,62 | 1,469 | 6,461E-04 |
| Pygb | brain glycogen phosphorylase | 2441,94 | 2449,73 | 1702,18 | 1667,96 | 1,469 | 2,988E-06 |
| Cited2 | Cbp/p300-interacting transactivator, with Glu/Asp-rich carboxy-terminal domain, 2 | 9808,29 | 8973,66 | 5951,31 | 6203,94 | 1,446 | 9,875E-03 |
| Strn | striatin, calmodulin binding protein | 1222,19 | 1104,21 | 795,73 | 765,32 | 1,443 | 4,545E-02 |
| Kif1bp | KIF1 binding protein | 1861,92 | 1708,83 | 2240,97 | 2152,46 | 0,794 | 2,425E-05 |
| Acyp1 | acylphosphatase 1, erythrocyte (common) type | 266,01 | 217,34 | 298,92 | 308,51 | 0,704 | 3,227E-02 |
| Bre | brain and reproductive organ-expressed protein | 1185,28 | 1034,99 | 1455,61 | 1473,40 | 0,702 | 1,320E-02 |
| Ets2 | E26 avian leukemia oncogene 2, 3' domain | 3870,34 | 3892,71 | 6108,01 | 5583,63 | 0,697 | 2,631E-03 |
| Klhl21 | kelch-like 21 | 2262,13 | 2282,62 | 3345,76 | 3323,90 | 0,687 | 6,509E-06 |
| Rab3gap1 | RAB3 GTPase activating protein subunit 1 | 1684,55 | 1748,98 | 2644,72 | 2583,49 | 0,677 | 1,396E-03 |
| Sra1 | steroid receptor RNA activator 1 | 1461,46 | 1206,53 | 1895,28 | 1809,95 | 0,667 | 1,036E-02 |
| Spats2 | spermatogenesis associated, serine-rich 2 | 1999,70 | 2099,58 | 3237,54 | 3281,51 | 0,640 | 4,658E-22 |
| Bbs5 | Bardet-Biedl syndrome 5 (human) | 256,95 | 257,72 | 419,88 | 407,78 | 0,632 | 1,516E-05 |
| Lrch1 | leucine-rich repeats and calponin homology (CH) domain containing 1 | 735,49 | 790,16 | 1287,48 | 1251,32 | 0,631 | 7,705E-03 |
| Gpt2 | glutamic pyruvate transaminase (alanine aminotransferase) 2 | 2455,46 | 2402,01 | 4134,33 | 3973,49 | 0,605 | 1,633E-03 |
| Nudt13 | nudix (nucleoside diphosphate linked moiety X)-type motif 13 | 211,47 | 201,05 | 355,35 | 335,88 | 0,599 | 1,988E-06 |
| Pou4f1 | POU domain, class 4, transcription factor 1 | 78,06 | 76,88 | 125,67 | 130,04 | 0,591 | 3,997E-03 |
| Slc20a2 | solute carrier family 20, member 2 | 2233,50 | 2360,74 | 3857,69 | 4058,20 | 0,582 | 4,750E-11 |
| Zrsr1 | zinc finger (CCCH type), RNA binding motif and serine/arginine rich 1 | 370,85 | 367,07 | 651,98 | 639,06 | 0,574 | 6,688E-07 |
| Pik3cb | phosphatidylinositol 3-kinase, catalytic, beta polypeptide | 508,28 | 521,91 | 928,63 | 911,20 | 0,573 | 1,892E-06 |
| Klf9 | Kruppel-like factor 9 | 492,81 | 679,96 | 1082,50 | 1231,55 | 0,552 | 1,840E-02 |
| Ptgr2 | prostaglandin reductase 2 | 896,37 | 849,58 | 1650,33 | 1571,38 | 0,541 | 1,238E-12 |
| Exoc6 | exocyst complex component 6 | 350,55 | 356,96 | 627,73 | 663,94 | 0,538 | 2,348E-09 |
| Maf | avian musculoaponeurotic fibrosarcoma (v-maf) AS42 oncogene homolog | 576,41 | 515,54 | 1040,29 | 961,36 | 0,536 | 1,637E-03 |
| Ubl3 | ubiquitin-like 3 | 1503,41 | 1377,91 | 2649,86 | 2605,53 | 0,529 | 3,157E-18 |
| Aktip | thymoma viral proto-oncogene 1 interacting protein | 1201,69 | 1136,22 | 2329,88 | 2190,49 | 0,519 | 1,793E-07 |
| Ttc9 | tetratricopeptide repeat domain 9 | 1710,17 | 1663,20 | 3341,14 | 3252,98 | 0,511 | 7,044E-32 |
| Pkdcc | protein kinase domain containing, cytoplasmic | 194,75 | 196,01 | 386,31 | 389,24 | 0,504 | 1,219E-05 |
| Cd34 | CD34 antigen | 1285,15 | 1291,30 | 2407,74 | 2567,88 | 0,503 | 1,401E-16 |
| Cds2 | CDP-diacylglycerol synthase (phosphatidate cytidylyltransferase) 2 | 3244,14 | 3286,20 | 6775,39 | 6903,59 | 0,476 | 5,560E-07 |
| Ttll11 | tubulin tyrosine ligase-like family, member 11 | 266,37 | 229,93 | 518,06 | 486,00 | 0,473 | 1,023E-05 |
| Rcan2 | regulator of calcineurin 2 | 679,10 | 737,32 | 1468,43 | 1568,83 | 0,470 | 3,164E-10 |
| Fjx1 | four jointed box 1 (Drosophila) | 12,67 | 18,04 | 45,53 | 40,85 | 0,442 | 8,889E-03 |
| Usp20 | ubiquitin specific peptidase 20 | 1153,94 | 1101,09 | 2683,26 | 2651,07 | 0,415 | 3,388E-26 |
| Tpd52l1 | tumor protein D52-like 1 | 39,48 | 41,66 | 109,78 | 101,66 | 0,410 | 1,822E-04 |
| Tom1 | target of myb1 homolog (chicken) | 14,29 | 10,95 | 37,30 | 27,06 | 0,405 | 2,574E-03 |
| Creld1 | cysteine-rich with EGF-like domains 1 | 1031,91 | 898,85 | 2402,85 | 2238,65 | 0,402 | 6,734E-16 |
| D8Ertd82e | DNA segment, Chr 8, ERATO Doi 82, expressed | 189,36 | 260,61 | 678,41 | 652,76 | 0,399 | 1,376E-06 |
| Agl | amylo-1,6-glucosidase, 4-alpha-glucanotransferase | 1796,73 | 1872,06 | 4833,24 | 4946,82 | 0,378 | 3,020E-09 |
| Cilp2 | cartilage intermediate layer protein 2 | 17,24 | 17,09 | 60,42 | 47,02 | 0,364 | 2,639E-02 |
| Htr7 | 5-hydroxytryptamine (serotonin) receptor 7 | 31,07 | 38,25 | 140,85 | 105,73 | 0,362 | 5,886E-10 |
| Adamtsl4 | ADAMTS-like 4 | 553,65 | 468,89 | 1288,08 | 1309,40 | 0,358 | 5,643E-14 |
| Slc40a1 | solute carrier family 40 (iron-regulated transporter), member 1 | 156,36 | 143,24 | 563,25 | 464,09 | 0,309 | 8,486E-11 |
| Herc3 | hect domain and RLD 3 | 312,13 | 340,88 | 1082,82 | 1116,97 | 0,305 | 2,257E-29 |
| Klf5 | Kruppel-like factor 5 | 608,12 | 728,85 | 2330,35 | 2416,86 | 0,302 | 3,134E-06 |
| Mid1 | midline 1 | 288,64 | 366,50 | 1191,58 | 1267,15 | 0,289 | 3,561E-04 |
| Pcx | pyruvate carboxylase | 497,34 | 464,49 | 1759,70 | 1622,82 | 0,286 | 7,055E-14 |
| Bmp4 | bone morphogenetic protein 4 | 340,82 | 253,08 | 1029,21 | 952,91 | 0,266 | 6,044E-37 |
| Ak5 | adenylate kinase 5 | 19,33 | 12,26 | 51,92 | 47,98 | 0,256 | 9,465E-05 |
| Acacb | acetyl-Coenzyme A carboxylase beta | 22,33 | 19,93 | 76,94 | 84,42 | 0,236 | 1,093E-05 |
| Atp1b1 | ATPase, Na+/K+ transporting, beta 1 polypeptide | 506,46 | 451,93 | 1830,61 | 1918,65 | 0,236 | 1,019E-29 |
| Akap6 | A kinase (PRKA) anchor protein 6 | 1105,42 | 1221,02 | 5153,86 | 5420,53 | 0,225 | 9,017E-09 |
| 5730508B09Rik | RIKEN cDNA 5730508B09 gene | 62,73 | 56,16 | 255,98 | 254,53 | 0,221 | 8,784E-20 |
| Zbtb8b | zinc finger and BTB domain containing 8b | 1,04 | 1,31 | 8,28 | 6,27 | 0,210 | 4,612E-02 |
| Foxq1 | forkhead box Q1 | 23,55 | 24,38 | 136,08 | 116,56 | 0,209 | 6,645E-15 |
| 1700112E06Rik | RIKEN cDNA 1700112E06 gene | 9,88 | 9,47 | 47,51 | 49,40 | 0,192 | 1,783E-04 |
| Fam179a | family with sequence similarity 179, member A | 1,94 | 2,30 | 14,05 | 14,23 | 0,162 | 2,262E-04 |
| Egln3 | egl-9 family hypoxia-inducible factor 3 | 1367,42 | 1378,25 | 8156,85 | 8976,48 | 0,154 | 6,702E-34 |
| Axin2 | axin2 | 22,54 | 28,18 | 187,84 | 236,37 | 0,119 | 7,261E-20 |
| Cd3g | CD3 antigen, gamma polypeptide | 0,69 | 0,79 | 9,45 | 6,84 | 0,115 | 2,794E-02 |
| Scml4 | sex comb on midleg-like 4 (Drosophila) | 138,60 | 128,36 | 1162,46 | 1116,27 | 0,115 | 3,862E-42 |
| Colec10 | collectin sub-family member 10 | 11,28 | 13,78 | 112,93 | 127,66 | 0,108 | 2,017E-22 |
| Tmem117 | transmembrane protein 117 | 25,94 | 31,16 | 296,11 | 315,69 | 0,099 | 4,650E-12 |
| Bfsp2 | beaded filament structural protein 2, phakinin | 0,69 | 0,79 | 10,25 | 9,64 | 0,082 | 1,912E-04 |
| Serpina12 | serine (or cysteine) peptidase inhibitor, clade A (alpha-1 antiproteinase, antitrypsin), member 12 | 0,17 | 1,15 | 18,07 | 19,52 | 0,059 | 2,798E-07 |
| Pak6 | p21 protein (Cdc42/Rac)-activated kinase 6 | 0,17 | 0,26 | 4,03 | 4,83 | 0,055 | 9,128E-03 |
| Scel | sciellin | 5,42 | 5,00 | 95,09 | 91,96 | 0,054 | 1,210E-03 |
| Shisa2 | shisa family member 2 | 24,82 | 25,41 | 503,15 | 504,00 | 0,050 | 2,435E-07 |
| Gcnt1 | glucosaminyl (N-acetyl) transferase 1, core 2 | 19,57 | 20,32 | 611,46 | 571,83 | 0,036 | 1,410E-38 |
| Cap2 | CAP, adenylate cyclase-associated protein, 2 (yeast) | 121,35 | 123,45 | 4446,18 | 4502,92 | 0,027 | 5,170E-28 |
| Hrasls | HRAS-like suppressor | 0,52 | 0,26 | 15,95 | 14,23 | 0,018 | 1,121E-07 |
| Mn1 | meningioma 1 | 3,04 | 1,78 | 116,83 | 107,90 | 0,016 | 6,534E-24 |
|  |  |  |  |  |  |  |  |
| **Nrf2-basal** | | | | | | | |
| Pcdh20 | protocadherin 20 | 25,06 | 26,30 | 2,05 | 0,54 | 48,470 | 3,203E-11 |
| Cr2 | complement receptor 2 | 6,72 | 8,65 | 0,76 | 0,31 | 28,347 | 2,267E-04 |
| Areg | amphiregulin | 684,12 | 593,39 | 67,61 | 62,84 | 9,443 | 6,897E-16 |
| Ppm1h | protein phosphatase 1H (PP2C domain containing) | 16,11 | 14,79 | 4,31 | 1,58 | 9,363 | 2,205E-02 |
| 5830416P10Rik | RIKEN cDNA 5830416P10 gene | 51,41 | 41,74 | 8,27 | 4,80 | 8,693 | 1,290E-09 |
| Hs3st5 | heparan sulfate (glucosamine) 3-O-sulfotransferase 5 | 22,92 | 28,36 | 3,48 | 3,55 | 7,996 | 2,875E-04 |
| Polq | polymerase (DNA directed), theta | 520,10 | 582,75 | 90,23 | 103,09 | 5,653 | 6,233E-18 |
| Klf12 | Kruppel-like factor 12 | 34,79 | 61,05 | 9,33 | 11,50 | 5,307 | 7,037E-03 |
| Rorb | RAR-related orphan receptor beta | 675,24 | 740,03 | 144,54 | 155,97 | 4,745 | 1,936E-16 |
| Tubb3 | tubulin, beta 3 class III | 7746,01 | 6379,35 | 1502,85 | 1422,03 | 4,486 | 2,117E-10 |
| Camk1d | calcium/calmodulin-dependent protein kinase ID | 292,64 | 383,84 | 61,79 | 86,02 | 4,462 | 2,143E-17 |
| Styk1 | serine/threonine/tyrosine kinase 1 | 48,33 | 44,10 | 11,69 | 10,26 | 4,299 | 1,321E-08 |
| Fgl2 | fibrinogen-like protein 2 | 95,05 | 98,16 | 26,68 | 26,47 | 3,708 | 1,626E-10 |
| Serpinb9b | serine (or cysteine) peptidase inhibitor, clade B, member 9b | 3590,20 | 3096,88 | 948,40 | 932,74 | 3,320 | 1,344E-43 |
| Sox13 | SRY (sex determining region Y)-box 13 | 184,81 | 185,40 | 58,03 | 59,90 | 3,095 | 3,257E-11 |
| Tube1 | epsilon-tubulin 1 | 302,67 | 321,59 | 82,77 | 109,66 | 2,933 | 1,305E-16 |
| Zwint | ZW10 interactor | 6787,31 | 6551,05 | 2198,22 | 2281,82 | 2,871 | 1,237E-13 |
| Aim1 | absent in melanoma 1 | 2111,41 | 2196,08 | 699,58 | 791,65 | 2,774 | 1,492E-11 |
| Khdrbs3 | KH domain containing, RNA binding, signal transduction associated 3 | 751,53 | 692,25 | 250,29 | 257,65 | 2,687 | 1,297E-13 |
| Cep290 | centrosomal protein 290 | 601,20 | 766,88 | 273,93 | 307,45 | 2,494 | 2,027E-03 |
| Frk | fyn-related kinase | 919,73 | 981,89 | 444,90 | 425,92 | 2,305 | 3,547E-17 |
| Arrdc4 | arrestin domain containing 4 | 34,00 | 27,02 | 13,76 | 11,93 | 2,265 | 2,857E-02 |
| Eya1 | eyes absent 1 homolog (Drosophila) | 602,21 | 693,50 | 317,60 | 307,30 | 2,257 | 7,007E-08 |
| Ddr2 | discoidin domain receptor family, member 2 | 4733,16 | 5870,75 | 2283,51 | 2634,16 | 2,229 | 1,459E-04 |
| Snrpd3 | small nuclear ribonucleoprotein D3 | 5094,00 | 4933,73 | 2453,20 | 2417,41 | 2,041 | 9,620E-06 |
| Btc | betacellulin, epidermal growth factor family member | 124,78 | 147,59 | 58,25 | 72,92 | 2,024 | 1,099E-03 |
| Tmtc3 | transmembrane and tetratricopeptide repeat containing 3 | 1224,90 | 1523,90 | 755,28 | 779,23 | 1,956 | 1,750E-02 |
| Mcm9 | minichromosome maintenance complex component 9 | 384,86 | 398,56 | 209,80 | 204,20 | 1,952 | 1,902E-10 |
| Bax | BCL2-associated X protein | 4143,47 | 3723,92 | 2009,97 | 1951,11 | 1,909 | 2,123E-04 |
| Rai14 | retinoic acid induced 14 | 6562,99 | 7252,87 | 4067,89 | 4038,60 | 1,796 | 1,022E-06 |
| Man2a1 | mannosidase 2, alpha 1 | 3791,56 | 4410,18 | 2264,34 | 2468,93 | 1,786 | 1,632E-03 |
| Zfp619 | zinc finger protein 619 | 51,34 | 70,42 | 36,58 | 39,68 | 1,775 | 2,085E-02 |
| Glis3 | GLIS family zinc finger 3 | 274,23 | 312,83 | 191,26 | 179,42 | 1,744 | 6,420E-03 |
| Nfyb | nuclear transcription factor-Y beta | 1230,97 | 1243,13 | 709,43 | 720,61 | 1,725 | 1,324E-09 |
| Sgk1 | serum/glucocorticoid regulated kinase 1 | 2364,17 | 2588,24 | 1448,03 | 1530,84 | 1,691 | 1,907E-03 |
| Rps12 | ribosomal protein S12 | 221,23 | 234,76 | 137,43 | 140,13 | 1,675 | 1,811E-02 |
| Zfp706 | zinc finger protein 706 | 5750,08 | 5583,72 | 3443,98 | 3360,53 | 1,662 | 2,364E-07 |
| Trib1 | tribbles homolog 1 (Drosophila) | 1091,92 | 1154,86 | 673,49 | 697,26 | 1,656 | 5,326E-06 |
| Cdk5rap2 | CDK5 regulatory subunit associated protein 2 | 2239,26 | 2342,49 | 1422,66 | 1426,84 | 1,642 | 4,833E-05 |
| Dusp1 | dual specificity phosphatase 1 | 1843,87 | 1605,46 | 1069,49 | 986,71 | 1,627 | 1,845E-05 |
| Plekha3 | pleckstrin homology domain-containing, family A (phosphoinositide binding specific) member 3 | 1459,13 | 1499,37 | 912,68 | 926,63 | 1,618 | 1,106E-09 |
| Prep | prolyl endopeptidase | 9558,26 | 9828,14 | 5997,29 | 6126,85 | 1,604 | 3,967E-12 |
| Fmnl2 | formin-like 2 | 572,70 | 762,59 | 471,88 | 479,47 | 1,590 | 1,774E-02 |
| Lnp | limb and neural patterns | 1278,96 | 1254,11 | 863,55 | 807,70 | 1,553 | 1,525E-08 |
| Slc41a2 | solute carrier family 41, member 2 | 1592,07 | 1525,24 | 1048,73 | 984,73 | 1,549 | 4,137E-04 |
| Kif2a | kinesin family member 2A | 2175,49 | 2291,93 | 1522,97 | 1485,45 | 1,543 | 1,353E-08 |
| Tmtc4 | transmembrane and tetratricopeptide repeat containing 4 | 738,85 | 827,98 | 558,05 | 536,96 | 1,542 | 3,611E-04 |
| Twf1 | twinfilin, actin-binding protein, homolog 1 (Drosophila) | 3823,77 | 3742,94 | 2580,15 | 2429,88 | 1,540 | 5,011E-10 |
| Lmna | lamin A | 58183,61 | 51119,34 | 34940,16 | 33275,87 | 1,536 | 2,713E-03 |
| Klf4 | Kruppel-like factor 4 (gut) | 3249,40 | 3227,39 | 2019,38 | 2107,99 | 1,531 | 8,600E-05 |
| Prrx1 | paired related homeobox 1 | 4913,95 | 5463,34 | 3441,64 | 3568,47 | 1,531 | 6,696E-07 |
| Tiam2 | T cell lymphoma invasion and metastasis 2 | 1925,37 | 1892,90 | 1321,20 | 1237,01 | 1,530 | 7,381E-03 |
| Zfp365 | zinc finger protein 365 | 1521,25 | 1632,86 | 1019,99 | 1077,14 | 1,516 | 3,997E-03 |
| Auts2 | autism susceptibility candidate 2 | 521,50 | 627,43 | 388,86 | 414,22 | 1,515 | 1,009E-02 |
| Nr2f2 | nuclear receptor subfamily 2, group F, member 2 | 980,23 | 969,91 | 650,13 | 645,30 | 1,503 | 7,322E-11 |
| Klf6 | Kruppel-like factor 6 | 6158,96 | 6421,87 | 4090,98 | 4276,69 | 1,502 | 6,330E-05 |
| 2210018M11Rik | RIKEN cDNA 2210018M11 gene | 783,99 | 945,87 | 646,05 | 631,62 | 1,498 | 4,228E-02 |
| Pla2g6 | phospholipase A2, group VI | 1017,65 | 1094,11 | 1511,91 | 1555,65 | 0,703 | 1,714E-05 |
| Apool | apolipoprotein O-like | 933,16 | 839,29 | 1232,77 | 1199,88 | 0,699 | 3,538E-02 |
| Col1a2 | collagen, type I, alpha 2 | 34147,79 | 32649,89 | 50236,89 | 47706,99 | 0,684 | 4,959E-04 |
| Tsc2 | tuberous sclerosis 2 | 2090,71 | 2200,71 | 3218,88 | 3235,91 | 0,680 | 6,694E-03 |
| Socs3 | suppressor of cytokine signaling 3 | 1029,02 | 908,13 | 1408,35 | 1343,83 | 0,676 | 1,013E-03 |
| Cdc42ep3 | CDC42 effector protein (Rho GTPase binding) 3 | 3779,59 | 3298,29 | 5135,45 | 4988,04 | 0,661 | 7,015E-06 |
| E2f6 | E2F transcription factor 6 | 1101,20 | 1087,71 | 1572,30 | 1650,21 | 0,659 | 5,333E-12 |
| St3gal4 | ST3 beta-galactoside alpha-2,3-sialyltransferase 4 | 1097,31 | 1040,00 | 1634,20 | 1591,94 | 0,653 | 1,052E-07 |
| Clasp1 | CLIP associating protein 1 | 3150,62 | 3565,21 | 5082,81 | 5498,67 | 0,648 | 3,361E-02 |
| Arfgap3 | ADP-ribosylation factor GTPase activating protein 3 | 3491,12 | 3108,28 | 4839,32 | 4857,49 | 0,640 | 6,205E-13 |
| Fam26e | family with sequence similarity 26, member E | 32,94 | 29,91 | 35,77 | 47,11 | 0,635 | 2,946E-02 |
| Fzd7 | frizzled homolog 7 (Drosophila) | 2107,82 | 2270,60 | 3727,40 | 3626,10 | 0,626 | 9,843E-06 |
| Lifr | leukemia inhibitory factor receptor | 1591,33 | 1732,00 | 2960,66 | 2824,11 | 0,613 | 1,458E-05 |
| Samd4 | sterile alpha motif domain containing 4 | 1763,06 | 1952,68 | 3203,14 | 3188,70 | 0,612 | 2,493E-03 |
| Ranbp6 | RAN binding protein 6 | 378,44 | 401,04 | 634,99 | 655,15 | 0,612 | 1,126E-04 |
| Cd164 | CD164 antigen | 12550,05 | 12622,26 | 20660,44 | 20667,43 | 0,611 | 3,027E-10 |
| Ttc33 | tetratricopeptide repeat domain 33 | 1134,50 | 1037,64 | 1759,49 | 1708,48 | 0,607 | 8,303E-07 |
| Zfp652 | zinc finger protein 652 | 986,09 | 1154,70 | 1873,87 | 1902,93 | 0,607 | 1,459E-02 |
| Mrc2 | mannose receptor, C type 2 | 3888,04 | 3829,63 | 6866,48 | 6358,26 | 0,602 | 4,092E-05 |
| Sorbs2 | sorbin and SH3 domain containing 2 | 3397,05 | 3321,08 | 5388,04 | 5522,72 | 0,601 | 2,877E-06 |
| Lmo7 | LIM domain only 7 | 4654,75 | 4891,59 | 7790,67 | 8215,12 | 0,595 | 6,367E-04 |
| Tcf4 | transcription factor 4 | 2525,57 | 2792,30 | 4978,16 | 4756,01 | 0,587 | 4,568E-04 |
| Pja2 | praja 2, RING-H2 motif containing | 3634,28 | 3728,24 | 6583,40 | 6542,45 | 0,570 | 2,308E-03 |
| St5 | suppression of tumorigenicity 5 | 1593,01 | 1535,79 | 2956,41 | 2727,64 | 0,563 | 9,329E-08 |
| Fchsd2 | FCH and double SH3 domains 2 | 520,21 | 577,77 | 1087,58 | 1091,35 | 0,529 | 9,184E-05 |
| Tmem44 | transmembrane protein 44 | 89,28 | 89,13 | 151,38 | 168,74 | 0,528 | 5,121E-05 |
| Sipa1l2 | signal-induced proliferation-associated 1 like 2 | 409,05 | 494,53 | 940,54 | 937,87 | 0,527 | 4,950E-05 |
| Serpinb9 | serine (or cysteine) peptidase inhibitor, clade B, member 9 | 675,57 | 687,51 | 1215,87 | 1309,76 | 0,525 | 3,925E-11 |
| Angpt1 | angiopoietin 1 | 2522,35 | 2308,64 | 4620,07 | 4433,68 | 0,521 | 4,326E-10 |
| Rtp4 | receptor transporter protein 4 | 48,56 | 34,34 | 60,83 | 67,12 | 0,512 | 3,232E-03 |
| Cdadc1 | cytidine and dCMP deaminase domain containing 1 | 479,45 | 493,53 | 1006,02 | 978,58 | 0,504 | 2,787E-21 |
| Abcb9 | ATP-binding cassette, sub-family B (MDR/TAP), member 9 | 16,38 | 12,46 | 30,16 | 24,79 | 0,503 | 1,500E-02 |
| Has2 | hyaluronan synthase 2 | 592,29 | 617,40 | 1313,86 | 1231,96 | 0,501 | 5,544E-04 |
| Kcnj2 | potassium inwardly-rectifying channel, subfamily J, member 2 | 324,04 | 433,66 | 892,13 | 895,20 | 0,484 | 8,086E-03 |
| Kremen1 | kringle containing transmembrane protein 1 | 6899,47 | 6230,03 | 13600,96 | 12949,57 | 0,481 | 2,407E-14 |
| Sync | syncoilin | 1166,29 | 1275,03 | 2617,62 | 2686,70 | 0,475 | 1,599E-03 |
| Fyn | Fyn proto-oncogene | 3939,76 | 3731,87 | 7896,65 | 7944,01 | 0,470 | 1,695E-20 |
| P4ha1 | procollagen-proline, 2-oxoglutarate 4-dioxygenase (proline 4-hydroxylase), alpha 1 polypeptide | 6377,30 | 6402,89 | 13615,35 | 13659,13 | 0,469 | 1,052E-21 |
| Igfbp7 | insulin-like growth factor binding protein 7 | 3680,83 | 3445,24 | 7240,48 | 7357,09 | 0,468 | 1,024E-08 |
| Lactb | lactamase, beta | 253,23 | 265,79 | 608,83 | 569,30 | 0,467 | 2,286E-09 |
| Maml2 | mastermind like 2 (Drosophila) | 171,25 | 175,76 | 398,08 | 380,01 | 0,463 | 4,533E-04 |
| Rgmb | repulsive guidance molecule family member B | 822,85 | 803,25 | 1632,44 | 1766,10 | 0,455 | 3,476E-06 |
| Fam43a | family with sequence similarity 43, member A | 61,38 | 72,21 | 154,94 | 159,52 | 0,453 | 3,304E-06 |
| Napb | N-ethylmaleimide sensitive fusion protein attachment protein beta | 162,80 | 162,42 | 362,89 | 359,52 | 0,452 | 4,105E-05 |
| Cacna2d1 | calcium channel, voltage-dependent, alpha2/delta subunit 1 | 2836,08 | 3009,13 | 7081,41 | 7174,41 | 0,419 | 1,869E-06 |
| Sspn | sarcospan | 1794,89 | 1793,64 | 4225,99 | 4296,36 | 0,417 | 4,126E-12 |
| Setbp1 | SET binding protein 1 | 141,70 | 153,27 | 396,61 | 368,14 | 0,416 | 1,555E-02 |
| Tpm1 | tropomyosin 1, alpha | 27187,53 | 24689,29 | 66654,44 | 62352,96 | 0,396 | 1,117E-35 |
| Osmr | oncostatin M receptor | 503,98 | 537,92 | 1357,38 | 1365,28 | 0,394 | 5,918E-08 |
| Col14a1 | collagen, type XIV, alpha 1 | 117,76 | 160,93 | 498,04 | 414,14 | 0,389 | 1,118E-03 |
| Col5a1 | collagen, type V, alpha 1 | 10269,24 | 9619,03 | 26295,94 | 24860,05 | 0,387 | 1,064E-11 |
| Qpct | glutaminyl-peptide cyclotransferase (glutaminyl cyclase) | 77,95 | 75,08 | 228,54 | 225,69 | 0,333 | 3,086E-16 |
| Gstm6 | glutathione S-transferase, mu 6 | 1,89 | 2,40 | 7,29 | 7,37 | 0,326 | 4,599E-02 |
| Pdzrn3 | PDZ domain containing RING finger 3 | 999,87 | 1024,81 | 3485,93 | 3372,55 | 0,304 | 2,628E-18 |
| Sfrp2 | secreted frizzled-related protein 2 | 185,82 | 172,67 | 693,17 | 640,74 | 0,269 | 2,076E-11 |
| Ephb3 | Eph receptor B3 | 864,80 | 782,70 | 3081,44 | 2944,34 | 0,266 | 1,710E-33 |
| Grk5 | G protein-coupled receptor kinase 5 | 152,43 | 154,29 | 533,62 | 582,46 | 0,265 | 1,500E-12 |
| Rgma | repulsive guidance molecule family member A | 478,20 | 473,67 | 1816,48 | 1835,99 | 0,258 | 3,572E-42 |
| Pdlim3 | PDZ and LIM domain 3 | 2681,70 | 2371,35 | 9805,55 | 9711,01 | 0,244 | 9,138E-26 |
| Adcy8 | adenylate cyclase 8 | 10,41 | 5,82 | 25,85 | 24,05 | 0,242 | 9,282E-05 |
| Bcl6 | B cell leukemia/lymphoma 6 | 583,35 | 626,04 | 2594,75 | 2612,58 | 0,240 | 1,949E-36 |
| Esr1 | estrogen receptor 1 (alpha) | 98,42 | 104,39 | 461,54 | 437,38 | 0,239 | 2,754E-14 |
| Apol6 | apolipoprotein L 6 | 23,29 | 20,88 | 88,08 | 91,39 | 0,228 | 9,870E-11 |
| Il15 | interleukin 15 | 24,00 | 25,04 | 114,81 | 115,74 | 0,216 | 7,113E-07 |
| Prkg1 | protein kinase, cGMP-dependent, type I | 28,67 | 36,09 | 144,80 | 173,25 | 0,208 | 1,894E-05 |
| Inhbb | inhibin beta-B | 720,89 | 644,96 | 3408,30 | 3223,89 | 0,200 | 6,731E-41 |
| Rspo2 | R-spondin 2 homolog (Xenopus laevis) | 2,71 | 2,47 | 12,62 | 12,72 | 0,194 | 1,480E-03 |
| 1600014C23Rik | RIKEN cDNA 1600014C23 gene | 0,66 | 0,89 | 4,18 | 4,62 | 0,192 | 4,604E-02 |
| Habp2 | hyaluronic acid binding protein 2 | 8,05 | 7,62 | 51,80 | 40,98 | 0,186 | 6,708E-09 |
| Nuak1 | NUAK family, SNF1-like kinase, 1 | 592,47 | 568,17 | 3281,90 | 3059,53 | 0,186 | 7,397E-47 |
| Clec1a | C-type lectin domain family 1, member a | 8,35 | 11,01 | 60,87 | 60,86 | 0,181 | 1,118E-06 |
| Grb10 | growth factor receptor bound protein 10 | 6,54 | 5,85 | 27,17 | 34,60 | 0,169 | 6,604E-06 |
| Enah | enabled homolog (Drosophila) | 563,42 | 612,89 | 3884,28 | 3685,86 | 0,166 | 1,328E-12 |
| Lmcd1 | LIM and cysteine-rich domains 1 | 98,22 | 99,20 | 624,38 | 605,52 | 0,164 | 1,293E-22 |
| Hist1h1c | histone cluster 1, H1c | 863,59 | 688,13 | 4666,14 | 4324,29 | 0,159 | 9,624E-96 |
| Cdkn1a | cyclin-dependent kinase inhibitor 1A (P21) | 8631,23 | 8302,26 | 57791,01 | 55664,46 | 0,149 | 6,975E-52 |
| Nalcn | sodium leak channel, non-selective | 3,20 | 3,09 | 29,39 | 21,46 | 0,144 | 1,293E-02 |
| Tex16 | testis expressed gene 16 | 2,41 | 1,32 | 6,31 | 9,30 | 0,142 | 1,251E-02 |
| Lama4 | laminin, alpha 4 | 29,70 | 31,29 | 194,63 | 237,80 | 0,132 | 3,529E-03 |
| Epm2a | epilepsy, progressive myoclonic epilepsy, type 2 gene alpha | 48,85 | 48,46 | 341,06 | 368,34 | 0,132 | 1,207E-28 |
| Adssl1 | adenylosuccinate synthetase like 1 | 674,96 | 609,82 | 7047,22 | 6876,57 | 0,089 | 8,162E-70 |
| Tmem179 | transmembrane protein 179 | 1,54 | 1,41 | 18,37 | 17,30 | 0,082 | 5,131E-05 |
| Tnfrsf11b | tumor necrosis factor receptor superfamily, member 11b (osteoprotegerin) | 10,83 | 6,81 | 99,46 | 97,94 | 0,069 | 1,829E-16 |
| Mrvi1 | MRV integration site 1 | 2,24 | 2,76 | 164,57 | 177,28 | 0,016 | 8,401E-39 |
| Eef1a2 | eukaryotic translation elongation factor 1 alpha 2 | 6,35 | 1,15 | 77,92 | 76,24 | 0,015 | 3,329E-10 |
| Bcl2l14 | BCL2-like 14 (apoptosis facilitator) | 0,36 | 0,26 | 6,85 | 17,54 | 0,015 | 1,516E-07 |
| Ppp1r3a | protein phosphatase 1, regulatory (inhibitor) subunit 3A | 0,52 | 1,84 | 215,02 | 252,35 | 0,007 | 2,267E-38 |
| ^a^Data are mean normalized reads (n = 3) as determined by RNA sequencing. | | | | | | | |
| ^b^Data are ratios of mean expression levels between infected myoblasts (Mb-Tg) versus infected myotubes (Mt-Tg). | | | | | | | |
| ^c^FDR-corrected *p*-value. | | | | | | | |
| ^d^Color code indicates relative differences in expression levels for each mRNA with red color indicating higher expression levels than blue color. | | | | | | | |

**Table S3.** Sequences of primers used in this study.

| mRNA | Accession No. | Forward primer | Reverse primer |
| --- | --- | --- | --- |
| MmActb | NM_007393 | 5’- GTACCACCATGTACCCAGGC -3’ | 5’- AAGGGTGTAAAACGCAGCTC -3’ |
| MmG6pdh2 | NM_019468 | 5’- AGGACCTACCCATGGTCACA -3’ | 5’- GGCAAGGAGGCTGTCCATAG -3’ |
| MmG6pdhx | NM_008062 | 5’- GAGAGTGGGCTTCCAGTACG -3’ | 5’- GGACAAAATGGCGGTCCAAG -3’ |
| Mm6Pgd | NM_001081274 | 5’- TGCCATGTGGACCAGACAAG -3’ | 5’- AGGCACACCCAGAGAGTGTA -3’ |
| MmPcx | NM_008797 | 5’- ATGACTCTGGAAGGCGACGA -3’ | 5’- GCCATTGCAGGTAGTGTGTG -3’ |
| MmGys1 | NM_030678 | 5’- CCGTGCCTTTCTTGGATTGC -3’ | 5’- GATTCGGGAAGCCACACAGA -3’ |
| MmPygm | NM_011224 | 5’- GAGTGGACACGGATGGTGAT -3’ | 5’- TAGAGACTGGGGAAAGTGGGT -3’ |
| TgBag1 | [TGME49_259020](http://toxodb.org/toxo/showRecord.do?name=TranscriptRecordClasses.TranscriptRecordClass&project_id=ToxoDB&source_id=TGME49_259020-t26_1&gene_source_id=TGME49_259020)* | 5’- AGCGGAGAAAGTGGACGATG -3’ | 5’- TGATTTGTTGCTTTGCGCCC -3’ |
| TgAct | TGME49_209030* | 5’- GGCGAACCGTGAGAGAATGA -3’ | 5’- GCGTCCAGCAAGATCCAAAC -3’ |

Mm: *Mus musculus*, Tg: *T. gondii*; *www.toxodb.org
